# The developmental hourglass model is applicable to the spinal cord based on single‐cell transcriptomes and non‐conserved *cis*‐regulatory elements

**DOI:** 10.1111/dgd.12750

**Published:** 2021-09-28

**Authors:** Katsuki Mukaigasa, Chie Sakuma, Hiroyuki Yaginuma

**Affiliations:** ^1^ Department of Neuroanatomy and Embryology School of Medicine Fukushima Medical University Fukushima Japan

**Keywords:** evolution, gene regulatory networks, Shh, spinal cord, the developmental hourglass model

## Abstract

The developmental hourglass model predicts that embryonic morphology is most conserved at the mid‐embryonic stage and diverges at the early and late stages. To date, this model has been verified by examining the anatomical features or gene expression profiles at the whole embryonic level. Here, by data mining approach utilizing multiple genomic and transcriptomic datasets from different species in combination, and by experimental validation, we demonstrate that the hourglass model is also applicable to a reduced element, the spinal cord. In the middle of spinal cord development, dorsoventrally arrayed neuronal progenitor domains are established, which are conserved among vertebrates. By comparing the publicly available single‐cell transcriptome datasets of mice and zebrafish, we found that ventral subpopulations of post‐mitotic spinal neurons display divergent molecular profiles. We also detected the non‐conservation of *cis*‐regulatory elements located around the progenitor fate determinants, indicating that the *cis*‐regulatory elements contributing to the progenitor specification are evolvable. These results demonstrate that, despite the conservation of the progenitor domains, the processes before and after the progenitor domain specification diverged. This study will be helpful to understand the molecular basis of the developmental hourglass model.

## Graphical abstract

We have demonstrated by comparing the single‐cell transcriptomes between mice and zebrafish that molecular profiles of the post‐mitotic spinal neurons are divergent. In addition, we found that *cis*‐regulatory elements located around the progenitor fate determinants, such as Pax6, Dbx2, and Olig2, are not conserved among vertebrates, indicating the rewiring of the upstream gene regulatory network. This developmental divergence of the spinal cord is in accordance with the developmental hourglass model.

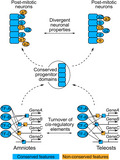

## 1 INTRODUCTION

The vertebrate neural tube can generate a diverse array of neurons in a precisely controlled and reproducible manner. During neurogenesis and neuronal differentiation, each neuron is assigned a distinct feature, such as neurotransmitter phenotype, axonal projection pathway, and cell body localization (Lai et al., 2016; Lu et al., 2015; Sagner & Briscoe, 2019). The initial phase of this process is the fate specification of progenitor cells, whereby molecularly defined progenitor domains are established with sharp boundaries along the dorsoventral axis of the neural tube (Briscoe et al., 2000). Each progenitor identity is specified through a highly complex gene regulatory network (GRN), which consists of a graded Shh signaling activity localized ventrally, Bmp and Wnt signaling molecules expressed dorsally, pan‐neural transcriptional activator Sox1–3, and a number of domain‐specific transcription factors (TFs) that function as repressors (Andrews et al., 2019; Balaskas et al., 2012; Delás & Briscoe, 2020; Kutejova et al., 2016; Nishi et al., 2015; Oosterveen et al., 2012; Peterson et al., 2012; Sagner & Briscoe, 2019; Zagorski et al., 2017). As the output of the GRN, 11 progenitor domains, termed dp1–6 in the dorsal half, and p0, p1, p2, pMN, and p3 in the ventral half, are established (Lai et al., 2016; Lu et al., 2015; Sagner & Briscoe, 2019). Subsequently, multiple neuronal subtypes are generated from a single progenitor domain. For example, V2a, V2b, and V2c interneurons (INs) are differentiated from the p2 domain (Del Barrio et al., 2007; Li et al., 2005; Panayi et al., 2010; Peng et al., 2007), and dI1i and dI1c INs are differentiated from the dp1 domain (Ding et al., 2012; Wilson et al., 2008). Each subtype of post‐mitotic neurons proceeds to the maturation process, such as migration, axonal projection, and circuit formation. The knowledge described above is mainly derived from chick and mouse studies, while a number of studies using zebrafish have demonstrated that the spatial architecture of the progenitor domains in the neural tube is largely conserved among vertebrates (Cheesman & Eisen, 2004; Cheesman et al., 2004; Gribble et al., 2007; Guner & Karlstrom, 2007; Lewis et al., 2005; Park et al., 2002; Schäfer et al., 2005).

However, there are overt differences between amniotes and teleosts with regard to post‐mitotic neuronal maturation. For example, V2a INs, which are defined by the expression of Vsx2 (Chx10), contribute to the locomotor rhythm generation in zebrafish (Eklöf‐Ljunggren et al., 2012), whereas V2a INs in mice play a role in the left–right alternation of limbs by providing excitatory input to the commissural INs (Crone et al., 2008, 2009). This implies that, during post‐mitotic differentiation in mice and zebrafish, V2a INs are assigned different properties or are placed in different positions within the spinal locomotor circuit (Kiehn, 2016). Another example is Robo3, an axon guidance receptor that is essential for commissural axons to cross the midline (Friocourt & Chédotal, 2017; Marillat et al., 2004; Sabatier et al., 2004). In the spinal cord of amniotes, Robo3 is expressed in V0, V1, and V3 INs in the ventral spinal cord, which encompass commissural INs (Friocourt et al., 2019; Tulloch et al., 2019). However, in zebrafish, although the double labeling of *robo3* and neuronal subtype markers has not been conducted, *robo3* expression is observed in the region encompassing motor neurons (MNs; Challa et al., 2005). If zebrafish MNs express *bona fide robo3*, the role of Robo3 has diverged between amniotes and zebrafish, as the MNs of amniotes never express Robo3 (Friocourt et al., 2019; Tulloch et al., 2019).

In addition to post‐mitotic differentiation, the process before the establishment of progenitor domains also differs between amniotes and zebrafish. *Shh* is an essential gene for the specification of the ventral neural tube and is expressed in the notochord and floor plate in amniotes (Echelard et al., 1993; Riddle et al., 1993; Roelink et al., 1994). In contrast, three *Shh*‐related genes, *shha* (*sonic hedgehog*), *shhb* (*tiggy winkle hedgehog*), and *ihhb* (*echidna hedgehog*), are expressed in the notochord and/or floor plate in zebrafish, conferring a functional redundancy of hedgehog (Hh) signaling (Currie & Ingham, 1996; Ekker et al., 1995; Krauss et al., 1993). The Hh signaling pathway culminates in Gli family TFs, which function as transcriptional activators or repressors, depending on the Hh signaling activity (Briscoe & Thérond, 2013). Amniotes possess three *Gli* genes (*Gli1*, *Gli2*, and *Gli3*), while teleosts possess four *Gli* genes (*gli1*, *gli2a*, *gli2b*, and *gli3*). Loss‐of‐function experiments of *Gli* genes led to clearly different phenotypes between mice and zebrafish. For example, *Gli1* knockout mice are viable and appear normal, showing that *Gli1* is not essential for embryogenesis in mice (Park et al., 2000). In contrast, *gli1*‐mutant zebrafish displayed severe cranial MN deficiency and reduced Hh‐target genes, such as *ptch1* and *nkx2.2a*, and died at the larval stage (Chandrasekhar et al., 1999; Karlstrom et al., 1996, 2003). In *Gli2* knockout mice, the floor plate has not been specified, and MNs aberrantly occupy the ventral‐most domain in the spinal cord, although MN itself is differentiated (Ding et al., 1998; Matise et al., 1998). In zebrafish, *gli2a* is completely dispensable for normal embryogenesis and growth to adulthood (Karlstrom et al., 2003; Vanderlaan et al., 2005; Wang et al., 2013), and *gli2b* knockdown causes a marked reduction of MNs in the spinal cord (Ke et al., 2008). Knockdown of *gli3* in zebrafish leads to a reduction of MNs (Vanderlaan et al., 2005); however, in *Gli3* mutant mice, patterning defects of the floor plate and MNs have not been observed (Persson et al., 2002).

To summarize the conservation and divergence of spinal cord development between amniotes and teleosts, the dorsoventral arrangement of the progenitor domains is well conserved, while the processes before and after the progenitor domain specification have diverged. This pattern of developmental divergence is highly consistent with the developmental hourglass model, which argues that embryonic morphology at the early and late developmental stages is divergent and that at the mid‐embryonic stage is conserved (Duboule, 1994; Hu et al., 2017; Irie & Kuratani, 2014). However, the process of spinal cord development has never been investigated from the perspective of this model. Herein, we provided evidence that the developmental hourglass model is applicable to spinal cord development. We examined the publicly available single‐cell transcriptome data from mice and zebrafish (Delile et al., 2019; Farnsworth et al., 2020), and provided other examples of diverse differentiation of post‐mitotic neurons. We also examined the transcriptional regulatory elements in the neural tube patterning genes based on chromatin immunoprecipitation sequencing (ChIP‐seq) and assay for transposase‐accessible chromatin using sequencing (ATAC‐seq) data, and sequence conservation, suggesting that *cis*‐regulatory elements contributing to the progenitor domain specification had undergone turnover (nucleotide changes) during vertebrate evolution. Based on our findings and the robust development of neuronal progenitor specification (Balaskas et al., 2012; Delás & Briscoe, 2020; Exelby et al., 2021; Xiong et al., 2013; Zagorski et al., 2017), we propose that the progenitor domain configuration in the neural tube is less evolvable owing to its canalization (Waddington, 1942, 1957).

## 2 MATERIALS AND METHODS

### 2.1 Experimental animals

Fertilized chicken eggs were obtained from Takeuchi farm (Nara, Japan) and incubated at 38℃ in a humidified incubator. Embryos were staged according to the Hamburger–Hamilton (HH) stage series (Hamburger & Hamilton, 1951). Mouse ICR strains were purchased from Japan SLC Inc (Japan). All animal experiments were performed in accordance with the Rules of Fukushima Medical University Animal Experiments, with the approval of the Animal Experiments Committee of Fukushima Medical University (approval number 2020103).

### 2.2 Single‐cell RNA‐seq data analysis

Mouse spinal cord single‐cell RNA‐seq (scRNA‐seq) data (Delile et al., 2019) were obtained from ArrayExpress (accession number E‐MTAB‐7320). The unique molecular identifier (UMI) count matrix was generated using Cell Ranger version 3.1.0 (10x Genomics). In this step, normalization was skipped to maximize sensitivity (cellranger aggr was executed with –normalize = none). The output matrix was fed into the Seurat (R package) version 3.1.5 (Butler et al., 2018). Zebrafish whole‐embryo scRNA‐seq data (Farnsworth et al., 2020) were obtained as Seurat object (.rds file) from Dr. Miller’s website (https://www.adammillerlab.com/resources‐1). Detailed procedures of normalization, data subsetting, graph‐based clustering, and dimensionality reduction using Seurat are available at https://github.com/kmukaigasa/Spinalcord_MouseZebrafish.

### 2.3 ChIP‐seq data analysis

ChIP‐seq data were obtained from the NCBI Sequence Read Archive (SRA). The accession numbers are as follows: GSE66961 for Pax6 (Sun et al., 2015); GSE42132 for Sox2 and Gli1 (Peterson et al., 2012); GSE61673 for Gli3, Nkx2‐2, Nkx6‐1, and Olig2 (Nishi et al., 2015); GSE114172 for Neurog2 (Aydin et al., 2019); GSE87180 for Pax7 (Mayran et al., 2018). The cell types used were embryonic forebrain (Pax6), AtT‐20 cell (Pax7), and neural cells differentiated from mouse embryonic stem (ES) cells (Sox2, Gli1/3, Nkx2‐2, Nkx6‐1, Olig2, and Neurog2). Sequencing adaptors and low‐quality bases were trimmed using Trimmomatic (version 0.39). FastQC (version 0.11.9) was used for sequence quality check. Read mapping to the mouse reference genome (mm10) was performed using Bowtie2 (version 2.3.5) with default settings. Reads with low mapping quality (MAPQ < 10) were removed using samtools (version 1.9). Peak calls were performed using MACS2 (version 2.2.6). The output bedGraph file was converted to BigWig format using bdg2bw (https://gist.github.com/jl32587/34370c995460f9d5ad65). The BigWig track was visualized in the UCSC genome browser.

### 2.4 ATAC‐seq data analysis

ATAC‐seq data (Metzis et al., 2018) were obtained from ArrayExpress (E‐MTAB‐6337). Data on the neural progenitor cells with spinal cord identity were utilized from the dataset. Read quality control was done as ChIP‐seq. Read mapping was performed using Bowtie2 with the following parameters: ‐X 2000 ‐‐sensitive‐local. PCR duplicates were marked using Picard (version 2.9.2). Reads with low mapping quality (MAPQ < 30) were removed by samtools. Peak calls were performed using MACS2 with the following parameters: ‐f BAMPE ‐g mm ‐q 0.05 ‐‐nomodel ‐‐keep‐dup auto ‐B.

### 2.5 Comparison of genomic sequences by VISTA

For interspecies comparisons of genomic sequences, partial genomic sequences were obtained from the UCSC genome browser (https://genome.ucsc.edu) and Ensembl (https://www.ensembl.org/index.html), and VISTA (Frazer et al., 2004) was used to align and visualize the results.

### 2.6 Vector construction

For RNA probe preparation, partial fragments of chick *ROBO3*, chick *SIM1*, chick *IRX3*, chick *DBX2*, and chick *GSX1* were amplified by PCR from chick embryonic spinal cord cDNA, and partial fragments of mouse *Robo3* and mouse *Sim1* were amplified from mouse embryonic spinal cord cDNA. The primer sequences used are presented in Table S2. The fragments were inserted into *pCR‐XL‐TOPO* (Thermo Fisher) or *pBluescriptKS*. For the reporter assay of putative *cis*‐regulatory module (CRMs), genomic DNA fragments were amplified by PCR from a solution that was prepared by digesting the tail tip of a C57BL/6J mouse. The amplified fragments were inserted into the *pSF‐pA‐MinProm‐eGFP* vector (Oxford Genetics). The positions of the CRMs within the mouse reference genome are provided in Table S3. For the construction of *pCAGGS‐mCherry*, the *mCherry* gene cassette of *pmCherry‐C1* (Clontech) was inserted into *pCAGGS* (Niwa et al., 1991).

### 2.7 In ovo electroporation

A small window was opened on top of the fertilized chicken eggshell. The electrodes were placed on both sides of the neural tube of an HH12‐13 chick embryo. Plasmid DNA was injected into the neural tube. During injection, electric pulses (25 V, 50 ms, five times, 950 ms interval) were applied using CUY21EDIT (BEX). The concentrations of the electroporated plasmids are listed in Table S5.

### 2.8 Immunohistochemistry

Embryos were fixed in 0.1 M phosphate buffer/4% paraformaldehyde (PFA) at room temperature for 45–60 min or 4℃ overnight. The fixed embryos were then cryoprotected in 20% sucrose at 4℃ overnight, embedded in an OCT compound, and cryosectioned. In cases of overnight fixed samples, sections were boiled for 20 min in sodium citrate buffer (10 mM sodium citrate, 0.05% Tween 20, pH 6.0) and cooled to room temperature. After washing with phosphate‐buffered saline (PBS) containing 0.1% Triton X‐100 (PBST), the sections were incubated with primary antibodies at 4℃ overnight, washed with PBST three times for 5 min, and incubated with secondary antibodies for 1–2 h at room temperature. After being washed with PBST three times for 5 min, the slides were coverslipped in VECTASHIELD (Vector Laboratories). The antibodies used are presented in Table S4. In the cases of anti‐Pax6 and anti‐Lhx1, Can Get Signal (TOYOBO, NKB‐401) was used as the diluent instead of PBST. Images were captured using an Olympus BX51 fluorescent microscope equipped with an Olympus DP71 digital camera or an Olympus FluoView FV1000 confocal microscope.

### 2.9 In situ hybridization

For double staining of immunohistochemistry and in situ hybridization, immunohistochemistry was performed as described above with primary and secondary antibody incubation for 1 h at room temperature, and the signal was developed using the VECTASTAIN Elite ABC kit (Vector Laboratories). Subsequently, the process of in situ hybridization commenced, as described below. The plasmid for the RNA probe template was linearized, and digoxigenin (DIG)‐labeled RNA probes were generated using a DIG RNA labeling mix (Sigma‐Aldrich, 11277073910) and T3 RNA polymerase (Promega, P2083).

Tissue slides were washed with PBS for 5 min, treated with proteinase K (2 µg/ml) for 10 min, washed with PBS for 5 min, fixed in 4% PFA for 10 min, and washed three times with PBS for 5 min. The slides were then incubated in an acetylation solution (100 mM triethanolamine, pH 8.0) twice for 2–3 min, transferred to a new acetylation solution, acetylated for 15 min by adding dropwise acetic anhydride (0.3% final concentration), and washed with PBS three times for 5 min. The slides were prehybridized with a hybridization buffer (50% formamide, 5× SSC, 5× Denhardt's solution, 500 µg/ml herring sperm DNA, and 250 µg/ml yeast RNA) for 2–3 h at room temperature, and hybridized with a hybridization buffer containing DIG‐labeled RNA probe. The slides were coverslipped and incubated in a humidified chamber at 70℃ overnight. The following day, the slides were transferred to 5× SSC at 70℃ for 5 min, washed twice in 0.2× SSC for 30 min at 70℃, and washed in 0.2× SSC for 30 min at room temperature. The slides were washed with buffer B1 (100 mM Tris‐HCl pH 7.5, 150 mM NaCl, 0.1% Tween 20) three times for 30 min, incubated with buffer B1 containing 10% heat‐inactivated normal goat serum for 2–3 h at room temperature, and then incubated with buffer B1 containing alkaline phosphatase conjugated anti‐DIG antibody (Roche 11093274910, 1:5000) and 1% heat‐inactivated normal goat serum at 4℃ overnight. The following day, the slides were washed five times with buffer B1 for 30 min, and incubated in buffer B3 (100 mM Tris‐HCl pH 9.5, 100 mM NaCl, 50 mM MgCl2, 0.1% Tween20) three times for 5 min. The slides were incubated with BM purple (Roche, 11442074001) until the signal was visualized (3 h to overnight). Next, the slides were washed three times with buffer B1 for 30 min, washed three times with PBS for 5 min, and coverslipped in Fluoromount (Diagnostic Biosystems, K024).

## 3 RESULTS

### 3.1 Two distinct subtypes of V3 INs in amniotes

**FIGURE 1:**
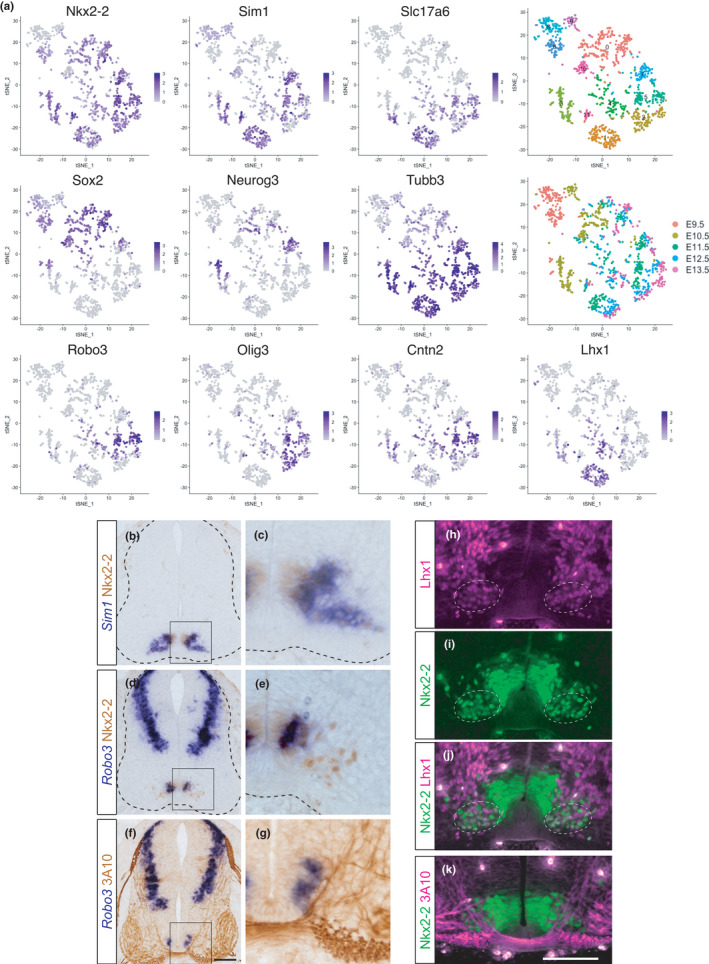
Two distinct subtypes of V3 INs in mice. (a) tSNE plot showing the cells with V3 IN identity derived from mouse embryonic spinal cords. The expression levels of the genes indicated are visualized on the tSNE plot. The top right panel shows the result of graph‐based clustering, with each cluster being colored differently. Cluster numbers (0–11) are labeled. The middle right panel shows the embryonic day when cells are corrected. (b–g) The expressions of Nkx2‐2, *Sim1*, and *Robo3* were examined in the mouse spinal cord at the forelimb level at E11.5. Growing axons were visualized by monoclonal antibody 3A10. Staining of in situ hybridization and immunohistochemistry was colored by blue and brown, respectively. c, e, and g show the enlarged views of the boxed areas in b, d, and f, respectively. The edges of the neural tube are demarcated by the broken lines. (h–j) Immunohistochemistry using Nkx2‐2 and Lhx1 antibodies. Nkx2‐2 and Lhx1 double‐positive cells are indicated by the broken‐line circles. (k) Immunohistochemistry using Nkx2‐2 and 3A10 antibodies. The ventral‐most region of the spinal cord is shown in h–k. Scale bar: 100 μm in f for b, d and f, and in k for h–k

To clarify whether post‐mitotic neuronal differentiation has diverged among vertebrates, we compared publicly available scRNA‐seq data from mice and zebrafish. We focused on V3 INs because we detected diversity in the gene expression among different species, as discussed below. First, we examined scRNA‐seq data obtained from the spinal cords of mouse embryos (Delile et al., 2019). The data consisted of the whole spinal cord cells from embryonic day (E) 9.5 to E13.5. We extracted data with V3 IN profiles (*Nkx2‐2* or *Sim1* positive), applied graph‐based clustering and dimensionality reduction by tSNE to these cells, and then identified the differentially expressed genes in each cluster (Table S1). The expression levels of several markers were visualized on the tSNE plots (Figure 1a). *Nkx2‐2* is a marker of the p3 progenitor domain and V3 INs (Briscoe et al., 1999). *Sim1* is a post‐mitotic V3 IN marker (Zhang et al., 2008). V3 INs are glutamatergic neurons, and thus express *Slc17a6* (*vGlut2*) (Zhang et al., 2008). The expression of *Sox2*, *Neurog3*, and *Tubb3* (*class III β‐tubulin*) in this order indicates the transition from progenitor to differentiated neurons (Carcagno et al., 2014). Thus, the medial‐to‐lateral direction in the spinal cord corresponds to the top‐to‐bottom direction in this plot. We found at least two distinct populations in V3 INs: one positive for *Robo3*, *Olig3*, and *Cntn2* (*Tag1*) (cluster 5 in Figure 1a), and the other positive for *Lhx1* (cluster 1 in Figure 1a, Table S1).

To distinguish these two subtypes in vivo, markers of each subtype (*Robo3* and Lhx1) were examined in the spinal cord of the mouse embryo at E11.5 (Figure 1b–k). Nkx2‐2 was used as a marker for the p3 progenitor domain and V3 INs. Post‐mitotic V3 INs were distinguished by *Sim1* expression. We found that the expression of *Robo3* was localized medially within post‐mitotic V3 INs. To confirm this further, commissural axons passing through and bisecting the V3 INs were utilized as landmarks. Some V3 INs were observed laterally to the commissural axons; however, these cells did not express *Robo3* (Figure 1f,g, and k). Rather, *Robo3* was selectively expressed by V3 INs located medially to the commissural axons (Figure 1f,g). In contrast, Lhx1 was expressed in the laterally located population of the V3 INs (Figure 1h–j). We performed the same examination using chick embryos and obtained identical results (Figure S1). These observations confirmed that there are at least two subtypes of V3 INs, which are transcriptionally, spatially, and probably also functionally distinct populations in the developing spinal cord in amniotes.

### 3.2 The gene expression profile of V3 IN in zebrafish was distinct from that in amniotes

**FIGURE 2:**
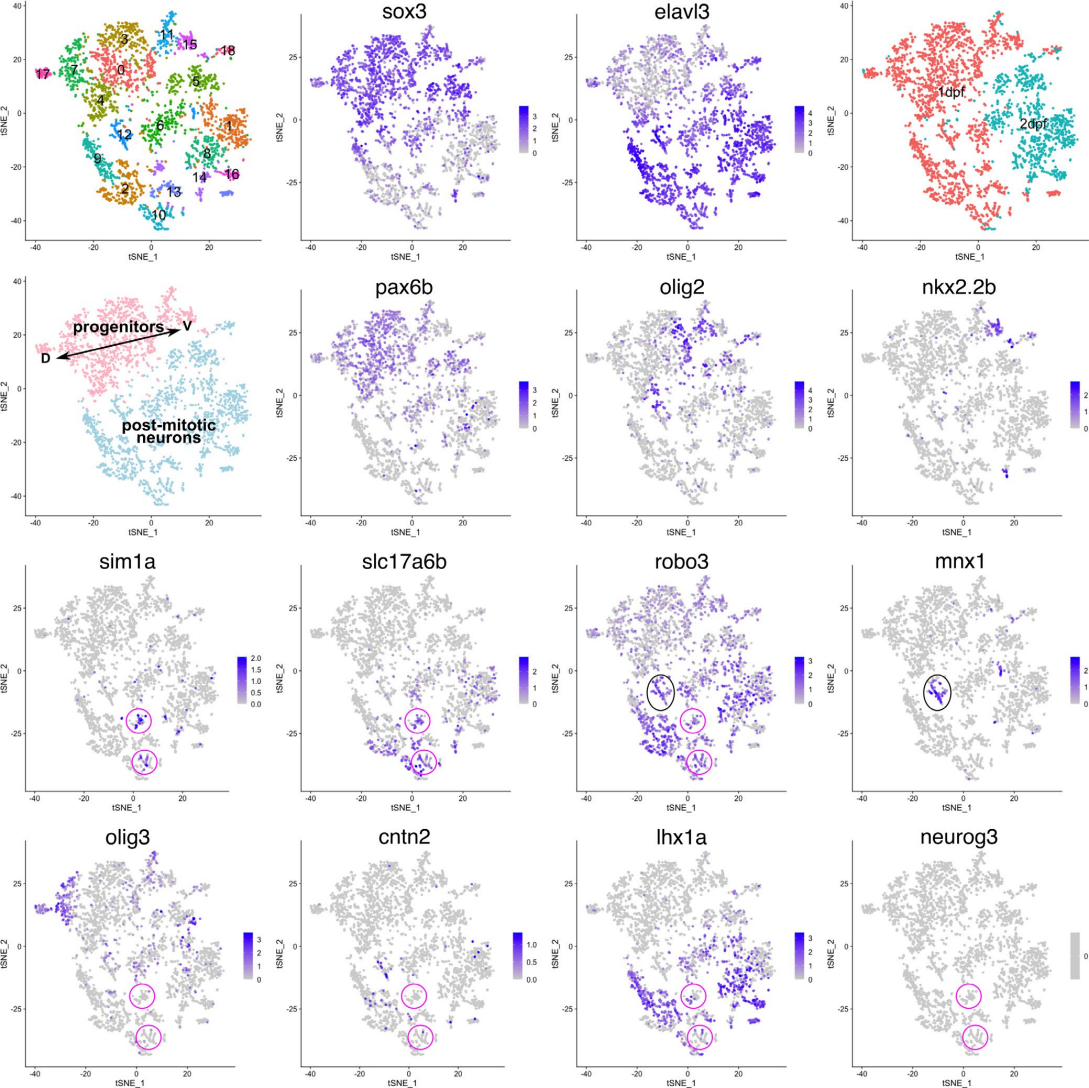
The gene expression profile of V3 IN in zebrafish is distinct from that in amniotes. tSNE plot showing the cells with spinal cord identity derived from zebrafish embryos (1 and 2 dpf). The top left panel shows the result of graph‐based clustering, with each cluster being colored differently. Cluster numbers (0–18) are labeled. The top right panel shows the embryonic day when the cells are corrected. The left‐most panel in the second row shows progenitors and post‐mitotic neurons with distinct colors. D and V indicate dorsal and ventral, respectively. The other panels show the expression levels of the indicated genes. The magenta and black circles indicate V3 INs and MNs, respectively

Next, we analyzed the scRNA‐seq data derived from zebrafish whole embryos at 1 and 2 days post‐fertilization (dpf; Farnsworth et al., 2020) to compare them with the gene expression profile of mouse V3 INs. We extracted the data with the spinal cord profiles (the detailed procedure of the data subsetting is provided in Figure S2). Clustering and dimensionality reduction were performed, in a similar manner to the data analysis of the mouse spinal cord (Figure 2). In the tSNE plot, the progenitor cells, which mainly consisted of 1 dpf cells, were identified by the expression of *sox3* and non‐expression of *elavl3* (*HuC*) (Figure 2, clusters 0, 3, 4, 7, 11, 15, and 17). The expression of domain‐specific TFs, such as *pax6b*, *olig2*, and *nkx2.2b*, was localized in a specific space in the tSNE plot, which was parallel to the expression domain in vivo along the dorsoventral axis of the neural tube (Figures 2 and S3). These data corroborate the evolutionary conservation of the progenitor domain organization in the neural tube (Cheesman & Eisen, 2004; Cheesman et al., 2004; Gribble et al., 2007; Guner & Karlstrom, 2007; Lewis et al., 2005; Park et al., 2002; Schäfer et al., 2005). V3 INs can be distinguished by the expression of *sim1a* (magenta circle in Figure 2). These V3 INs were *slc17a6b*‐positive glutamatergic neurons, like amniotes. However, unlike amniotes, almost all V3 Ins expressed *robo3* in zebrafish, and there was no expression of *olig3* and *cntn2* (Figures 2 and S4). The expression of *Lhx1* and *Robo3* was mutually exclusive in amniotes, yet this was not the case in zebrafish (Figures 1, 2, and S4). Furthermore, *neurog3*, one of the V3 IN‐specific TFs (Carcagno et al., 2014), was not expressed in the V3 INs of zebrafish (Figures 1 and 2). These results indicate that the V3 INs of zebrafish are not equivalent to those of amniotes as far as gene expression is concerned.

### 3.3 Distinct gene expression profiles in other ventral INs between mice and zebrafish

**FIGURE 3:**
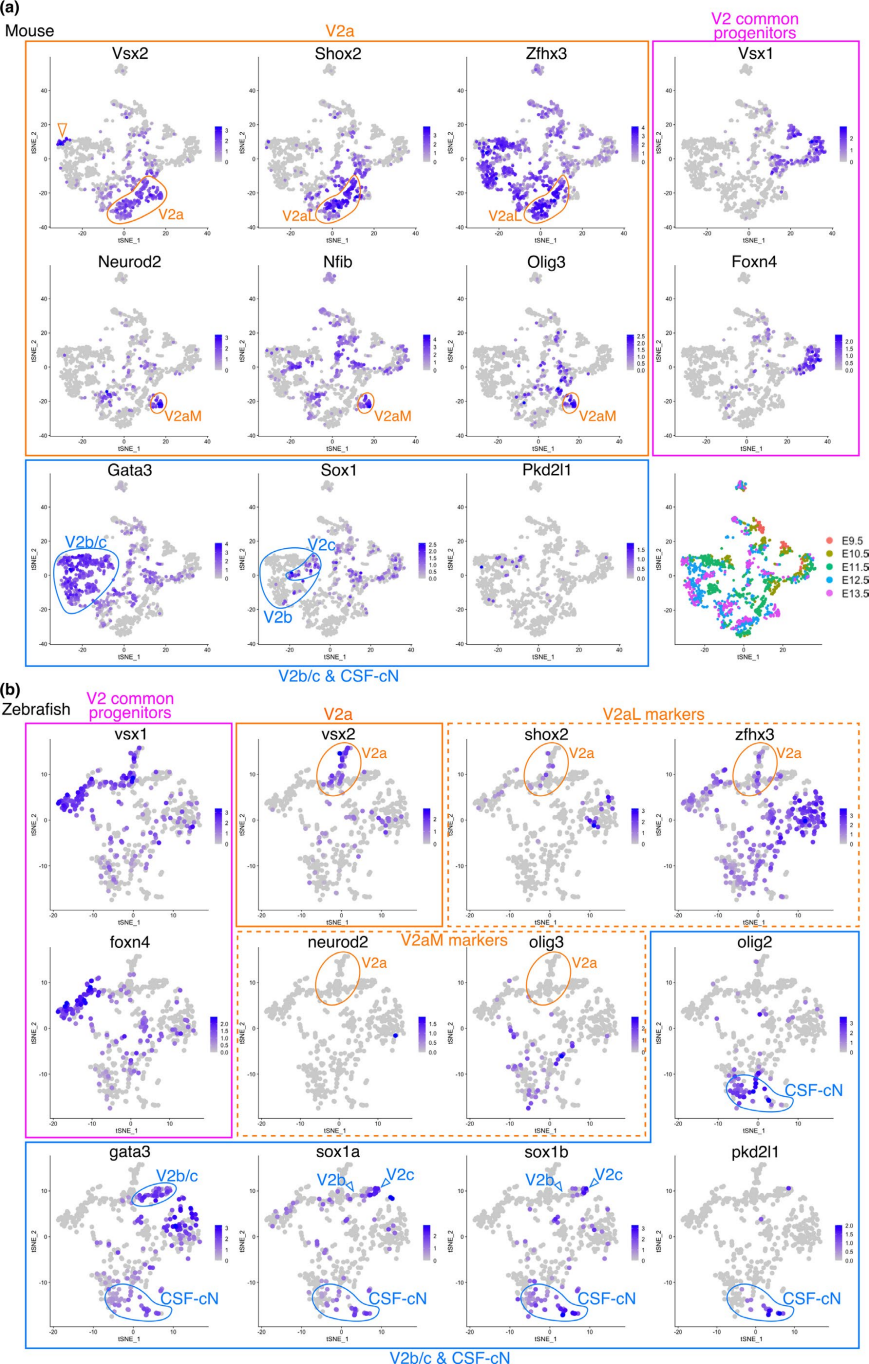
Distinct gene expression profiles in V2 INs between mice and zebrafish. tSNE plot showing the cells with V2 IN identity derived from mouse (a) and zebrafish (b) embryonic spinal cords. The expression levels of the indicated genes were visualized on the tSNE plot. Plots for V2 common progenitor markers are enclosed in magenta boxes. Plots for V2a IN markers are enclosed in yellow boxes. Plots for the V2b/c IN and CSF‐cN markers are enclosed in blue boxes. The bottom right panel in (a) shows the embryonic day when the cells are corrected. Within the tSNE plots, specific subpopulations (V2a, V2aM, V2aL, V2b, V2c, and CSF‐cNs) are indicated. The expression profiles of V2aL and V2aM markers (shox2, zfhx3, neurod2, and olig3) in zebrafish were different from those in mice (yellow broken‐line boxes in b). The Vsx2‐high minor population was found in the mouse tSNE plot as a distinct cluster (open arrowhead in a), which is likely to be type‐I V2a neurons, and other major V2a INs are probably type‐II neurons (Hayashi et al., 2018)

To determine whether the divergence of the gene expression profiles was evident only in V3 INs, we performed the same comparison focusing on V2 INs. We extracted cells with V2 IN identity (*Foxn4*, *Vsx1*, *Vsx2*, *Gata2*, or *Gata3* positive neurons) from the scRNA‐seq datasets, and compared the gene expression profiles in a manner similar to that employed for V3 INs. According to previous reports (Del Barrio et al., 2007; Hayashi et al., 2018; Kimura et al., 2008; Li et al., 2005; Panayi et al., 2010; Peng et al., 2007), we annotated five V2 IN subtypes on the mouse tSNE plot as follows: (1) V2 common progenitors (*Foxn4* ^+^, *Vsx1* ^+^); (2) V2a INs laterally enriched subtype (Hayashi et al., 2018) (*Vsx2* ^+^, *Shox2* ^+^, *Zfhx3* ^+^, referred to as V2aL); (3) V2a INs medially enriched subtype (Hayashi et al., 2018) (*Vsx2* ^+^, *Neurod2* ^+^, *Nfib* ^+^, *Olig3* ^+^, referred to as V2aM); (4) V2b INs (*Gata3* ^+^, *Sox1* ^–^); (5) V2c INs (*Gata3* ^+^, *Sox1* ^+^) (Figure 3a). In the zebrafish tSNE plot, we found that the V2 common progenitors and V2b/c INs corresponded to those of mice (Figure 3b, plots enclosed by magenta and blue boxes, respectively). However, unlike mouse V2a INs, the V2aL markers *shox2* and *zfhx3* were not colocalized, but rather mutually exclusive, and the V2aM markers *neurod2* and *olig3* were not expressed in the V2 INs in zebrafish (Figure 3b, plots enclosed by yellow broken‐line boxes). Furthermore, another V2aM marker, *Nfib*, could not be found in the zebrafish genome. These results suggest that the gene expression profiles of V2a INs have also diverged between mice and zebrafish.

The expression of Gata2/3 marks not only V2b/c INs but also cerebrospinal fluid‐contacting neurons (CSF‐cNs) in the vertebrate spinal cord, and CSF‐cNs can be distinguished from V2b/c INs by their Pkd2l1 expression (Djenoune et al., 2014; Petracca et al., 2016). CSF‐cNs were identified as a distinct cluster in zebrafish tSNE plots (Figure 3b, *gata3* ^+^, *pkd2l1* ^+^, *sox1a/b* ^+^), while in the mouse tSNE plots, there were few CSF‐cNs, which were scattered and did not make any recognizable clusters, suggesting that CSF‐cNs were not yet fully differentiated in mice at E13.5 (Figure 3a, plot for Pkd2l1). Indeed, amniotes’ CSF‐cNs are born at a significantly late phase of spinal cord development (E14 or later in mice), namely when gliogenesis commences (Petracca et al., 2016). In contrast, the CSF‐cNs of zebrafish are born early, that is, simultaneously with other INs and MNs (Park et al., 2004; Shin et al., 2007). This heterochronic development of CSF‐cNs is another example of the divergence of neuronal development in the vertebrate spinal cord (Petracca et al., 2016). We also examined the gene expression profiles of V0 (*Evx1/2* ^+^) and V1 (*En1* ^+^) INs briefly, which were suggestive of divergent gene expression profiles in V0 and V1 INs between mice and zebrafish (details are provided in Figure S5). Taken together, these results suggest that the divergence of gene expression profiles is not a specific feature of V3 INs; rather, it is a generally observed feature of the ventral INs of the spinal cord.

### 3.4 CRM in the Robo3 locus is not conserved in teleosts

**FIGURE 4:**
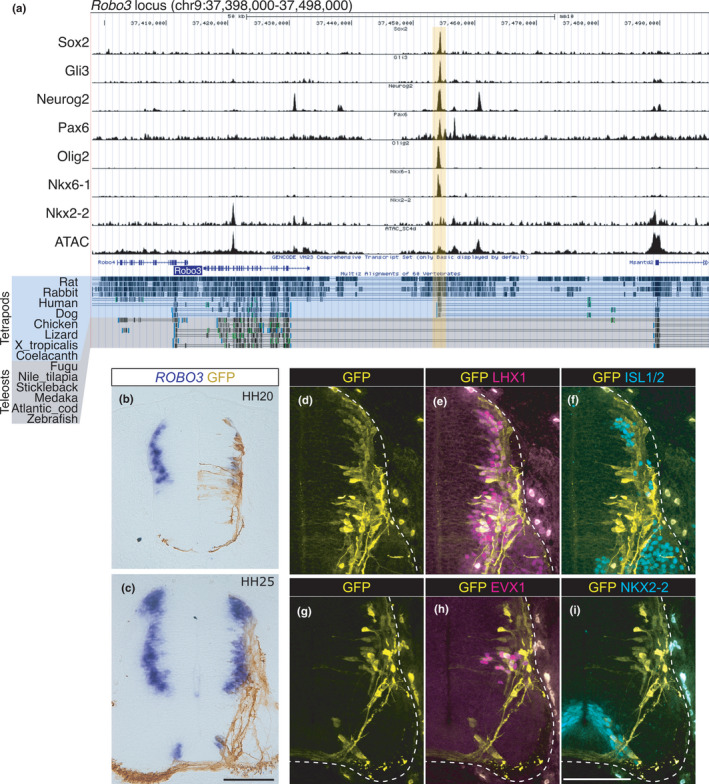
The CRM in the *Robo3* locus is not conserved in teleosts. (a) ChIP‐seq and ATAC‐seq peak call results are displayed in the UCSC genome browser with Multiz Alignments track. The *Robo3* locus in the mouse genome (mm10) is displayed. The region harboring multiple TF binding sites (*Robo3‐CRM*) is highlighted in yellow. Multiz Alignment tracks of tetrapods and teleosts are highlighted by different colors. (b–i) *Robo3‐CRM::GFP* was electroporated into chick neural tubes. (b, c) The expression of GFP and *ROBO3* was examined at HH20 (b) and HH25 (c) after electroporation. (d–i) The expression of the GFP and TFs indicated was examined at HH25 after electroporation. The edges of the neural tube are demarcated by the broken lines. The embryo numbers examined in the electroporation experiments are provided in Table S5. Scale bar: 100 μm in c for b and c, in i for d–i

As is shown in Figure 2 (black circle), *mnx1*‐positive MNs expressed *robo3* in zebrafish. This is clearly different from amniotes, in which *Robo3* is never expressed in MNs (Friocourt et al., 2019), implying that the transcriptional regulation of *Robo3* has diverged during vertebrate evolution. To confirm this, we searched for the transcription regulatory elements around the *Robo3* locus using the available ChIP‐seq and ATAC‐seq data of neural cells (Figure 4). We found a putative *cis*‐regulatory module (CRM) harboring multiple TF binding sites in an open chromatin state at approximately 20 kb upstream of the *Robo3* transcription start site (TSS; highlighted in yellow in Figure 4a; CRM in the *Robo3* locus is abbreviated as *Robo3‐CRM*). To confirm the function of *Robo3‐CRM*, a reporter assay was performed using a chick in ovo electroporation system. We cloned *Robo3‐CRM* from the mouse genome into a vector containing minimal promoter and *GFP* (the resulting construct was named *Robo3‐CRM::GFP*). After the electroporation of *Robo3‐CRM::GFP*, GFP expression overlapped with the endogenous *ROBO3* expression in the chick spinal cord (Figure 4b,c), and GFP‐positive axons crossed the midline (Figure 4c). More specifically, we found that GFP was expressed in LHX1‐positive dorsal and intermediate INs (Figure 4d,e), EVX1‐positive V0 INs (Figure 4g,h), and NKX2‐2‐positive V3 INs (Figure 4g,i), but not in ISL1/2‐positive dI3 INs or MNs (Figure 4d,f), consistent with the endogenous *ROBO3*‐expressing cells (Tulloch et al., 2019). These results demonstrate that *Robo3‐CRM* can recapitulate the normal expression pattern of *Robo3* almost completely in the spinal cord.

Notably, when sequence conservation was checked using Multiz alignments in the UCSC genome browser (Blanchette et al., 2004) and VISTA (Frazer et al., 2004) among vertebrates, *Robo3‐CRM* was conserved in tetrapods, but not in teleosts (highlighted in yellow, comparing tracks labeled “Tetrapods” and “Teleosts” in Figure 4a). Weak conservation of *Robo3‐CRM* was observed in the spotted gar, a basal actinopterygian (Figure S6), suggesting that the common ancestor of bony vertebrates possesses *Robo3‐CRM*, which has been lost in the teleost lineage (Lee et al., 2011). These results suggest that the divergent expression patterns of *robo3* in zebrafish are likely due to the loss of *Robo3‐CRM*. In addition, the divergence of *Robo3‐CRM* suggests that a part of axon guidance mechanisms in post‐mitotic neurons might have diverged during vertebrate evolution.

### 3.5 Non‐conservation of Gli binding sites in Gli1 and Ptch1 loci in vertebrates

**FIGURE 5:**
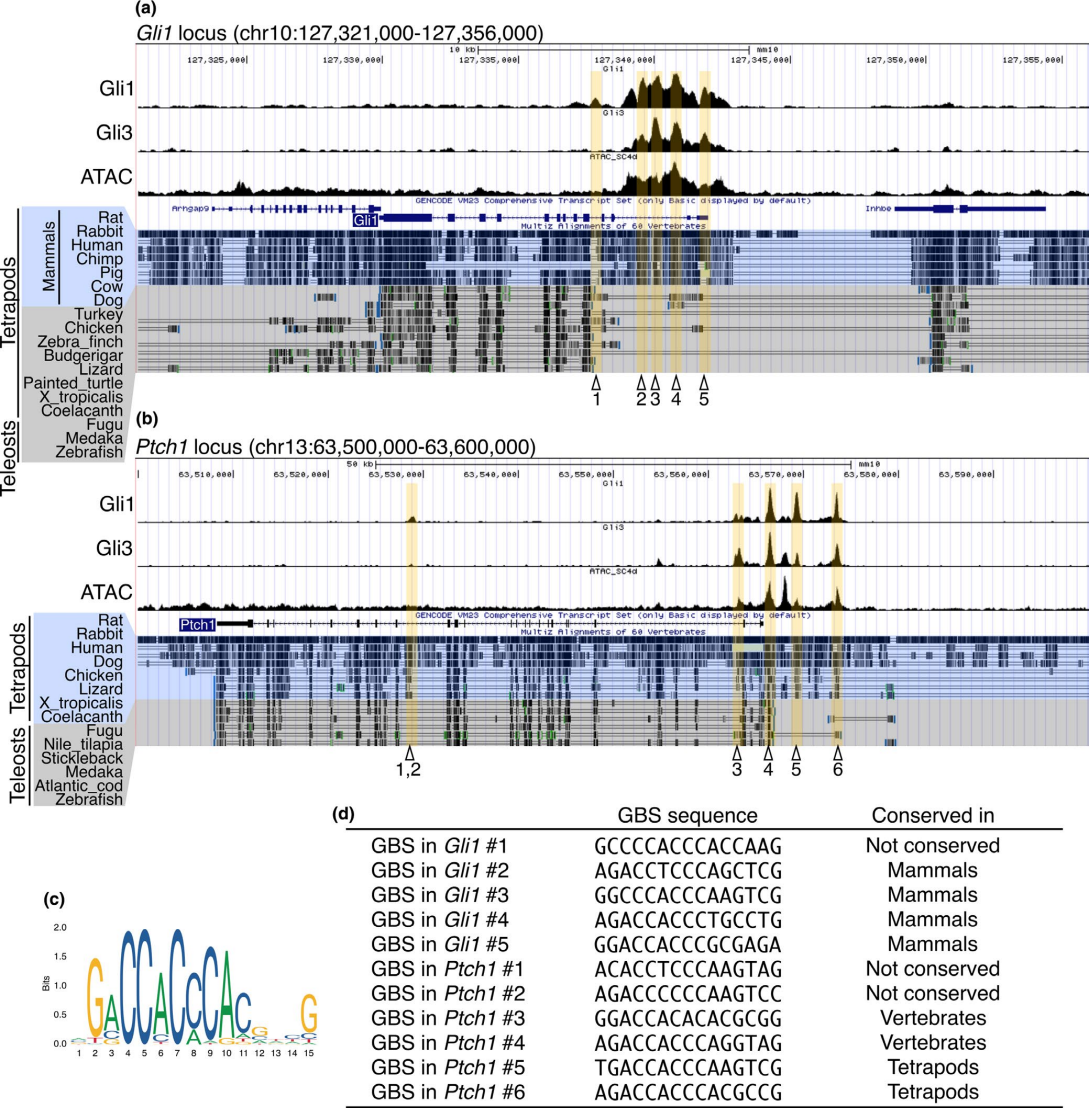
Non‐conservation of GBSs in *Gli1* and *Ptch1* loci in vertebrates. (a, b) Gli1/3 ChIP‐seq and ATAC‐seq peak call results are displayed in the UCSC genome browser with the Multiz Alignments track. The *Gli1* (a) and *Ptch1* (b) loci in the mouse genome (mm10) are shown. In the *Gli1* locus, the Multiz Alignment tracks of mammals and non‐mammals are highlighted by different colors. In the *Ptch1* locus, the Multiz Alignment tracks of tetrapods and teleosts are highlighted by different colors. The positions of GBSs are highlighted in yellow, and are indicated by sequential numbers in the *Gli1* and *Ptch1* loci independently. (c) The Gli binding motif represented by the sequence logo. (d) The DNA sequence of each GBS indicated in (a) and (b). The extent of conservation of each GBS is shown in the “Conserved in” column. More detailed conservation profiles are presented in Figure S7

Next, we examined whether the upstream process before the progenitor fate specification also diverged among vertebrates. Previous studies have reported that the impact of loss of Gli function on neuronal progenitors is different between mice and zebrafish (Chandrasekhar et al., 1999; Karlstrom et al., 2003; Tyurina et al., 2005; Vanderlaan et al., 2005; Wang et al., 2013). This raises the possibility that some Gli binding sites (GBSs), that is, the sites of action of Hh signaling, vary among vertebrates. To test this, *Gli1* and *Ptch1* loci were interrogated, as these genes are direct targets of Hh signaling. We searched for GBS in the mouse genome and checked sequence conservation using Multiz alignments in the UCSC genome browser and VISTA. Using Gli1 and Gli3 ChIP‐seq data, many ChIP‐seq peaks were observed in mouse *Gli1* and *Ptch1* loci near TSS and within introns (Figure 5a,b), confirming previous reports (Dai et al., 1999; Nishi et al., 2015). We scanned the genomic regions around the ChIP‐seq peaks and found five and six GBSs matching the Gli binding motif in *Gli1* and *Ptch1* loci, respectively (Figure 5c,d). ATAC‐seq data confirmed that most of these GBSs were in an open chromatin state in neural cells (Figure 5a,b, tracks labeled “ATAC”). We found that most of these GBSs were not conserved among vertebrates. In the case of *Gli1*, one GBS was found only in mice, and four other GBSs were conserved only in mammals (Figure 5a,d). In the *Ptch1* locus, two GBSs were conserved in vertebrates, but four others were not well conserved (Figure 5b,d and S7). These findings imply that the GBS is a highly flexible element.

### 3.6 Non‐conservation of the CRMs of progenitor fate specifying TFs in vertebrates

**FIGURE 6:**
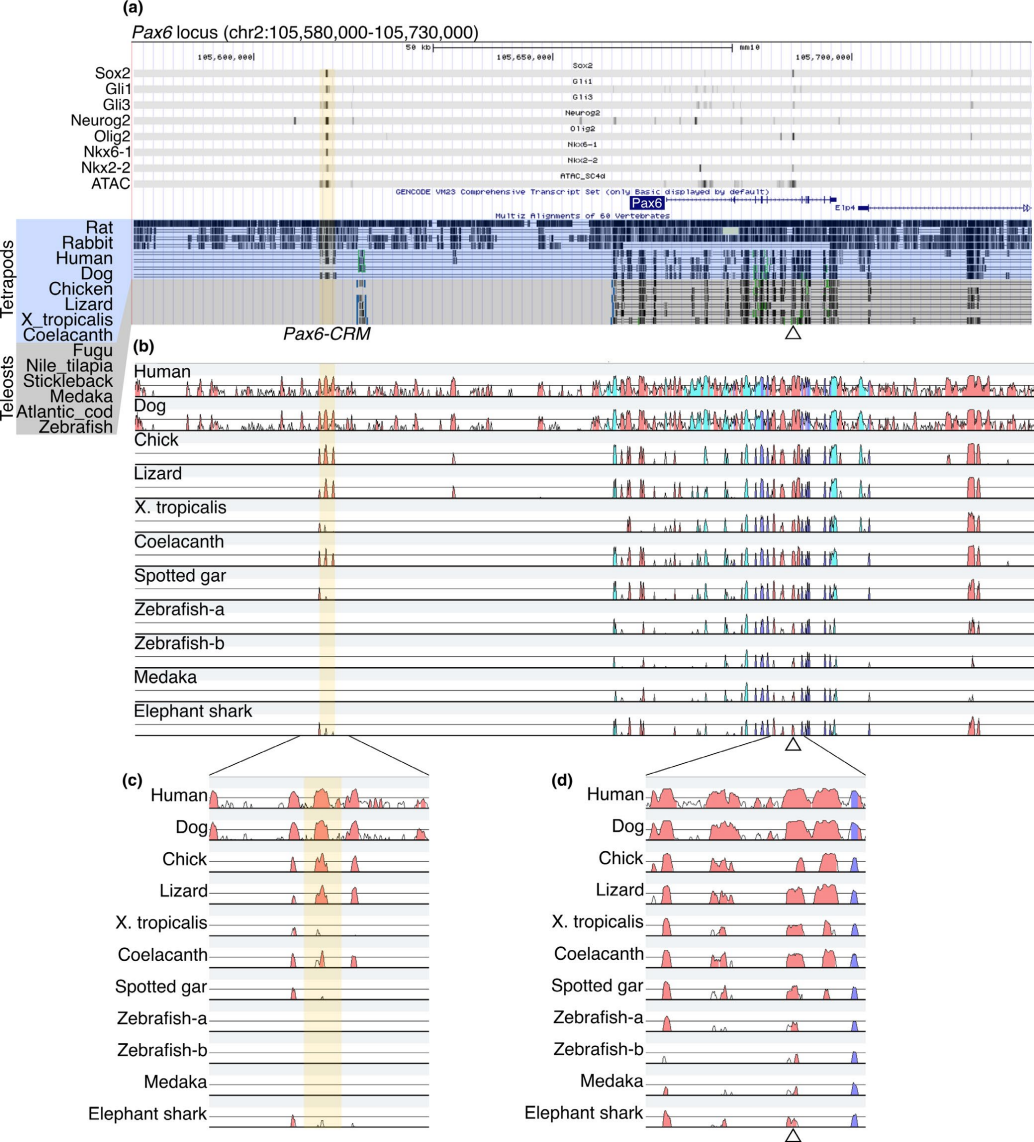
Identification of *Pax6‐CRM* and its diversification in vertebrates. (a) ChIP‐seq and ATAC‐seq peak call results are displayed in the UCSC genome browser with Multiz Alignments track (the display mode is dense). The mouse *Pax6* locus is displayed. Regions harboring multiple TF binding sites (*Pax6‐CRM*) are highlighted in yellow, and a previously validated CRM is indicated by an open triangle under the track (Oosterveen et al., 2012). (b) The same genomic regions from several species are aligned, and sequence conservation is visualized by VISTA. The base sequence is mouse, and the species compared are indicated on the left side. The peaks of the conserved regions are colored pink (noncoding sequences), dark blue (exons), or light blue (UTRs). Zebrafish possess two *pax6* genes (*pax6a* and *pax6b*); thus, both loci are included in the alignments (Zebrafish‐a and Zebrafish‐b correspond to the *pax6a* and *pax6b* loci, respectively). (c, d) Enlarged view of the plot focusing on CRMs. The previously identified CRM located in the intron is conserved in all species examined (d), whereas the *Pax6‐CRM* highlighted in yellow is not conserved in zebrafish and medaka (c)

As the regulatory elements in *Robo3*, *Gli1*, and *Ptch1* were not conserved among vertebrates, we expected that non‐conservation of the regulatory elements could be found in other genes. Thus, we applied the same analysis to TFs functioning as progenitor fate determinants expressed in the neural tube. Indeed, we found that *Pax6‐CRM*, *Gsx1‐CRM*, *Dbx2‐CRM*, *Irx3‐CRMs* (two CRMs, designated *Irx3‐CRM1* and *2*), and *Olig2‐CRMs* (two CRMs, designated *Olig2‐CRM1* and *2*) were conserved only in tetrapods, but were lost in teleosts (Figure 6 and Fig S8–S11, highlighted regions). These CRMs were in an open chromatin state in most cases, and were bound by Gli1, Gli3, Sox2, Neurog2, Pax6, Pax7, Olig2, Nkx2‐2, and Nkx6‐1 in various combinations (Figure 6 and Fig. S8–S11). These TFs are essential components of GRN and regulate the progenitor fate in the neural tube (Balaskas et al., 2012; Delás & Briscoe, 2020; Exelby et al., 2021; Kutejova et al., 2016). The non‐conservation of *Pax6‐CRM*, *Gsx1‐CRM*, *Dbx2‐CRM*, *Irx3‐CRMs*, and *Olig2‐CRMs* suggests that the *cis*‐regulatory elements contributing to the progenitor domain specification are not constrained despite the evolutionary conservation of the neural tube progenitor domains.

**FIGURE 7:**
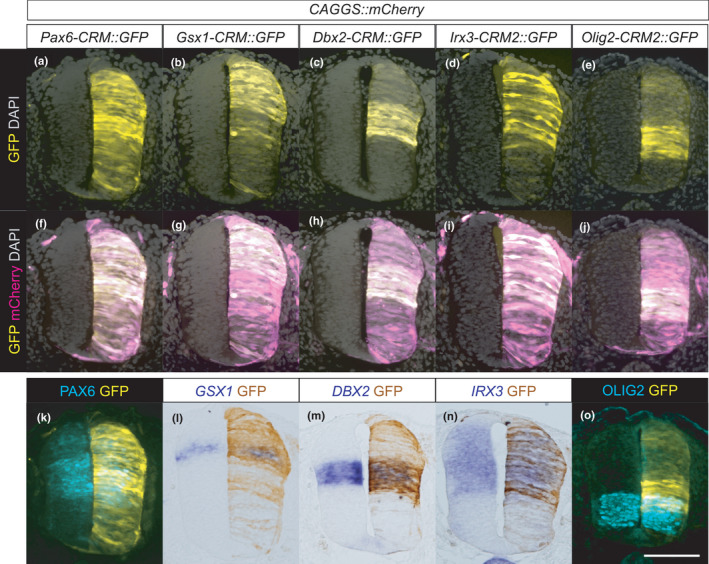
Enhancer functions of CRMs of progenitor fate‐specifying TFs. The CRM reporter vectors indicated were electroporated into chick neural tubes together with *CAGGS::mCherry* as a control vector. (a–j) The GFP and mCherry expression was examined at HH19–20 in the forelimb‐level neural tube. (k–o) The endogenous expression of the indicated genes was examined by in situ hybridization (l, m, n) or immunohistochemistry (k, o) together with the GFP expression. These five CRMs displayed enhancer functions, although the GFP expression domains incompletely overlapped with the endogenous expression domain. *Pax6‐CRM* induced GFP expression almost ubiquitously, but its expression was not observed in the roof plate (a, f, k). *Gsx1‐CRM* induced GFP expression in the dorsal neural tube, but not in the more ventral region than the endogenous expression domain (b, g, l). The GFP expression domains induced by *Dbx2‐CRM* or *Irx3‐CRM2* overlapped with the endogenous expression domain almost completely (c, d, h, i, m, and n). *Olig2‐CRM2* induced GFP expression in the intermediate to ventral region, which partially overlapped with, but was more dorsal than, the endogenous expression domain (e, j, and o). The number of embryos examined in the electroporation experiments is presented in Table S5. Scale bar: 100 μm

We identified several CRMs that were conserved only in tetrapods; however, except for *Olig2‐CRM1*, the in vivo functions of these CRMs have not been examined so far. Thus, we carried out a reporter assay by means of the chick in ovo electroporation system. We cloned the CRMs (*Pax6‐CRM*, *Gsx1‐CRM*, *Dbx2‐CRM*, *Irx3‐CRM1*, *Irx3‐CRM2*, and *Olig2‐CRM2*) from the mouse genome and constructed the GFP reporter vectors. Each reporter vector was electroporated at HH12–13, and GFP expression was examined at the stage when the progenitor domains were established (HH19–20). This experiment confirmed that *Pax6‐CRM* (Figure 7a,f and k), *Gsx1‐CRM* (Figure 7b,g and l), *Dbx2‐CRM* (Figure 7c,h and m), *Irx3‐CRM2* (Figure 7d,i and n), and *Olig2‐CRM2* (Figure 7e,j and o) function as enhancers in neural tube cells, although more regulatory elements are needed to precisely recapitulate the endogenous expression pattern. Note that this method cannot capture microRNA‐based translational repression, which indeed affects the dorsal boundary of Olig2 (Chen et al., 2011). The only exception was *Irx3‐CRM1*, which did not induce GFP expression after electroporation in the chick neural tube (data not shown, Table S5). The enhancer function of *Olig2‐CRM1* has been validated previously (Exelby et al., 2021; Oosterveen et al., 2012; Peterson et al., 2012; Wang et al., 2011). These reporter assay results confirmed the enhancer function of *Pax6‐CRM*, *Gsx1‐CRM*, *Dbx2‐CRM*, *Irx3‐CRM2*, and *Olig2‐CRMs* in amniotes. Nevertheless, these CRMs were lost in the teleosts. These findings indicate that the *cis*‐regulatory elements regulating gene expression in the neural tube are evolvable, while conserving the progenitor domain configuration.

## 4 DISCUSSION

### 4.1 Divergent properties of post‐mitotic neurons in vertebrates

First, in the present study, we compared the single‐cell transcriptomes of mice and zebrafish to elucidate the divergence of post‐mitotic neuronal differentiation. We found that, in amniotes, V3 INs can be divided into two distinct subtypes. One is located medially and expresses *Robo3*, *Olig3*, and *Cntn2*, and the other is located laterally and expresses *Lhx1* (Figure 1). We tried to make these V3 INs correspond with those of zebrafish, but were unsuccessful (Figures 1 and 2). Likewise, we also examined V2 INs and again found that the gene expression profiles of V2a INs differed between mice and zebrafish (Figure 3). This indicates that the spinal cord ventral INs of amniotes are not equivalent to those of zebrafish, at least regarding gene expression profiles, which supports the hypothesis that the properties of post‐mitotic neurons in the spinal cord have diverged among vertebrates. This is in contrast to the case of progenitor cells, in which each progenitor domain of zebrafish readily corresponds to that of amniotes (Figure S3).

The role of V3 INs in mice is to secure a stable locomotor rhythm (Zhang et al., 2008). Several studies have also suggested that V3 INs contribute to left–right synchronous motor output, such as gallop and bound (Danner et al., 2019; Kiehn, 2016; Rabe et al., 2009). These gaits can be expressed by quadrupeds, but not by fish. This suggests that the different locomotor behaviors between amniotes and teleosts are associated with the divergence of V3 IN properties. The same may be true for V2a INs, whose roles have diverged between amniotes and teleosts (Azim et al., 2014; Crone et al., 2008, 2009; Eklöf‐Ljunggren et al., 2012; Kiehn, 2016). Given that the number of muscles and corresponding MNs has significantly increased during tetrapod evolution, especially in limb muscles (Diogo & Abdala, 2010), additional layers of neuronal circuits are required to precisely control complex locomotor behavior (Kiehn, 2016). This may be accomplished, at least in part, by tinkering with the already existing neuronal population (Jacob, 1977), eventually leading to the divergence of post‐mitotic neuronal properties among vertebrates. Distinct expression profiles of *Robo3* between amniotes and zebrafish may be a strategy for neuronal tinkering (Figures 1, 2, and 4).

### 4.2 Limitations in cross‐species comparisons of single‐cell transcriptomes

Recently, several studies have reported cross‐species comparisons of single‐cell transcriptomes (Tosches et al., 2018; Zhu et al., 2018). To achieve a comprehensive and quantitative comparison of single‐cell transcriptomes, these studies integrated two datasets from distinct species after one‐to‐one ortholog identification and filtering out nonhomologous genes (Tosches et al., 2018; Zhu et al., 2018). In contrast, instead of integrating two datasets, in the present study, we analyzed the mouse and zebrafish scRNA‐seq datasets separately for the following two reasons. (1) Before the integration of the two transcriptome datasets, one‐to‐one ortholog identification is necessary. However, in the case of mice and zebrafish, this is difficult because of the whole genome duplication in teleost species. (2) In the current study, we focused on non‐conservation; thus, it would have been disadvantageous for us to filter out nonhomologous genes. Fortunately, an improved method of single‐cell transcriptome integration has recently been reported, which does not rely on one‐to‐one ortholog identification, but rather utilizes the weighted gene–gene homology graph, and can even detect paralog substitutions (Tarashansky et al., 2021). Such a new method may help overcome these difficulties in future studies.

In the present study, we used publicly available mouse and zebrafish scRNA‐seq data; however, these data were not fully comparable. The mouse dataset contained data for E9.5, 10.5, 11.5, 12.5, and 13.5 (Delile et al., 2019), while the zebrafish dataset contained data for 1, 2, and 5 dpf (Farnsworth et al., 2020; 5 dpf data were not included in our analysis, as that developmental stage is too advanced). Given the rapid embryogenesis of zebrafish, data that included more time points and shorter intervals would be favorable. In the case of zebrafish, data with spinal cord identity were extracted from whole embryonic data, based solely on the gene expression profiles, not on the anatomical data. Thus, theoretically, we cannot rule out the possibility that cells outside of the spinal cord are misannotated as the spinal cord, and vice versa. To further corroborate our hypothesis in future studies, it would be advisable to acquire new zebrafish data, obtained in shorter intervals during the neurogenic phase (for example, 6 h intervals during 12–48 dpf) in combination with the isolation of the spinal cord, by taking advantage of reporter transgenic lines and/or by manual dissection.

Despite these limitations, the results of previous studies are consistent with our hypothesis that post‐mitotic neuronal properties have diverged among vertebrates (Azim et al., 2014; Eklöf‐Ljunggren et al., 2012; Kiehn, 2016; Vigouroux et al., 2021; Zhu et al., 2018). For example, Zhu et al. (2018) reported that, even in closely related species (human and macaque), the neuronal transcriptomes in identical brain regions diverged between the two species.

### 4.3 Divergent processes leading to the conserved progenitor domains

In the latter part of this study, we investigated the transcriptional regulatory elements located around the progenitor fate specifying genes in order to reveal the extent to which the upstream process before the progenitor specification has diverged in vertebrates. In the *Gli1* locus, clustered GBSs near the TSS were conserved only in mammals, but not in teleosts (Figure 5). Even in birds and reptiles, these GBSs are conserved only partially. Nevertheless, in vertebrates, *Gli1* is commonly expressed in response to Hh signaling (Aglyamova & Agarwala, 2007; Karlstrom et al., 2003; Luo et al., 2006), suggesting the presence of distinct GBSs with equivalent functions. Indeed, zebrafish possess three GBSs in the *Gli1* locus, which are conserved only in some teleost species (Wang et al., 2013), indicating that regions where Hh signaling is eventually transduced has shifted during vertebrate evolution. This is supported by a similar situation in the *Ptch1* locus (Figure 5 and S7; Wang et al., 2013). These findings may partly explain the discrepancy in *Gli* loss‐of‐function phenotypes between mice and zebrafish.

It has been pointed out that many features of Shh are similarly observed in bicoid, a morphogen that plays a crucial role in patterning the anterior–posterior axis in *Drosophila* blastoderm (Briscoe & Small, 2015). It is noteworthy that bicoid binding sites have also undergone a rapid turnover in Diptera (McGregor et al., 2001). The flexibility of morphogen response elements might contribute to the integration of morphogen dependency into the patterning system of the embryo (Dearden & Akam, 1999; Miyamoto & Wada, 2013; Ren et al., 2020; Stauber et al., 1999).

**FIGURE 8:**
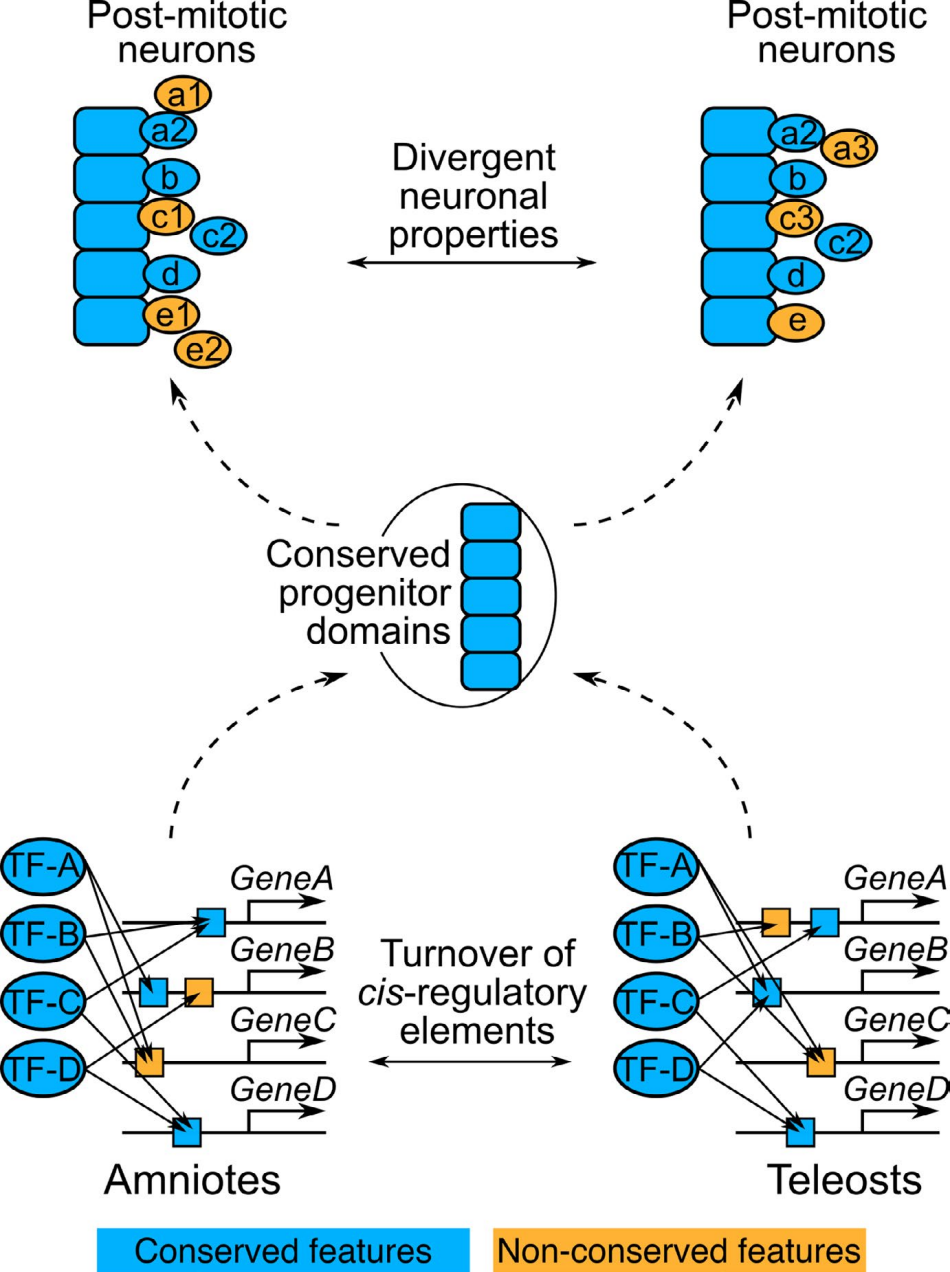
The hourglass‐like pattern of the developmental divergence of the spinal cord. A summary of this study is presented. In this drawing, development proceeds from the bottom to the top. Only five progenitor domains were set up for simplification. Conserved and non‐conserved features are colored in blue and orange, respectively. The bottom drawing represents GRNs regulating the progenitor domain establishment. CRMs (indicated by boxes) located in the progenitor fate specifying genes (*GeneA–D*) diverged (turnover) between amniotes and teleosts (orange boxes). Accordingly, these GRNs have been rewired. Nevertheless, these distinct GRNs result in the same progenitor domain organization. The top drawing represents the divergence of the differentiation process of post‐mitotic neurons. After individual cells leave the progenitor domains as post‐mitotic neurons (indicated by circles), some neurons undergo distinct maturation processes between amniotes and teleosts (orange circles). Thus, there exist neuronal subpopulations whose function is different between amniotes and teleosts

We identified functional CRMs bound by multiple TFs in the *Pax6*, *Gsx1*, *Dbx2*, *Irx3*, and *Olig2* loci in the mouse genome (Figures 6 and 7, Fig. S8–S11). These CRMs are conserved only in tetrapods, but are lost in teleosts (Figure 6, Fig. S8–S11). These findings indicate that, although the progenitor domain organization is conserved among vertebrates, the *cis*‐regulatory elements contributing to it are not constrained (Figure 8). This is considered a case of developmental system drift (DSD; True & Haag, 2001). Several studies have reported similar situations, in which divergent regulatory sequences in different species result in conserved gene expression (Barrière et al., 2012; Domené et al., 2013; Fisher et al., 2006; Hare et al., 2008; Ludwig et al., 1998, 2000; Paris et al., 2013; Stolfi et al., 2014; Swanson et al., 2011). In these cases, including the present study, the GRN architecture is likely to be maintained as a whole despite the *cis*‐element turnover, as demonstrated in mammalian evolution (Stergachis et al., 2014; Vierstra et al., 2014).

The process of neural tube formation differs between amniotes and zebrafish not only genetically but also morphogenetically. In amniotes, a midline groove is formed by the bending of the neural plate, the edges of which are then fused, thus forming a neural tube (Schoenwolf & Smith, 1990). On the other hand, in zebrafish, a solid neural keel with no central canal is formed by the convergent movement of the neural plate cells, and then the central canal opens secondarily (Schmitz et al., 1993). It follows that neural tube formation is a case of DSD from both genetic and morphogenetic viewpoints.

### 4.4 Molecular basis of the hourglass model in the spinal cord

Taken together, our results demonstrate that the divergence of the developmental process of the spinal cord is in accordance with the developmental hourglass model (Figure 8). What then imposes the hourglass‐like pattern on the evolution of spinal cord development? The progenitor regionalization in the neural tube is a highly robust developmental system and is thus insensitive to stochastic noises of graded morphogen activities and genetic mutations (Balaskas et al., 2012; Delás & Briscoe, 2020; Exelby et al., 2021; Xiong et al., 2013; Zagorski et al., 2017). Simulation studies have also proposed that developmental system robustness is an emergent property of the complex GRN (Bergman & Siegal, 2003; von Dassow et al., 2000; Siegal & Bergman, 2002). Although the authors of the aforementioned studies used in silico simulations and did not deal with neural tube development, their results indirectly support the robustness of the neuronal progenitor specification system, which is organized by highly complex GRN (Kutejova et al., 2016). From an evolutionary perspective, such a robust (canalized) developmental system can tolerate genetic mutations without phenotypic effects (Rutherford & Lindquist, 1998; Waddington, 1942, 1957). In other words, the progenitor specification system in the neural tube buffers genetic variations. We speculate that this situation consequently led to the turnover of *cis*‐regulatory elements, while the progenitor arrangement was maintained. Once cells exit and migrate away from the progenitor domains as post‐mitotic neurons, individual neurons depend on the mechanisms regulating post‐mitotic maturation, which is distinct from the progenitor specification GRN. As mentioned previously, the maturation process is considered to diverge in association with locomotor divergence. Eventually, through vertebrate evolution, the developmental divergence of the spinal cord might lead to an hourglass shape (Figure 8). The current study focused on the spinal cord. Thus, it is of particular interest to examine whether this scenario is also the case in other developmental systems or even at the whole embryonic level.

More importantly, if partial modification is permitted, neuroectodermal regionalization is conserved among bilaterians beyond vertebrates (Arendt, 2018; Denes et al., 2007; Jung & Dasen, 2015). This suggests that the developmental system robustness of neuroectodermal regionalization has already been acquired in the last common ancestor of bilaterians, and that upstream signals of the neuroectodermal regionalization had been modified after the divergence of phyla (e.g., the ventralization factor is dl in *Drosophila* or Shh in vertebrates; Cheesman et al., 2004; Cornell & von Ohlen, 2000; von Ohlen & Doe, 2000). This is supported by the notion of the common origin of the central nervous system (CNS) in bilaterians (Arendt, 2018; Arendt et al., 2016; Denes et al., 2007), but is inconsistent with the convergent evolution of the CNS (Martín‐Durán et al., 2018). Supporting the common evolutionary origin of the bilaterian CNS, we suggest the following evolutionary scenario. The trunk nerve cord, which develops from the regionalized neuroectoderm, has already been acquired in the common ancestor of bilaterians, and its development was so canalized that upstream regulators could be modified, while conserving the regionalized neuroectoderm (e.g., Hh signaling recruitment in deuterostomes; Miyamoto & Wada, 2013; Ren et al., 2020). Therefore, distinct upstream regulators of neuroectoderm regionalization among bilaterians can be considered to be DSD that had taken place during more than 500 million years of bilaterian evolution.

## Supporting information

Supplementary Material

Table S1
