# Supplementary material for: The developmental hourglass model is applicable to the spinal cord based on single‐cell transcriptomes and non‐conserved cis‐regulatory elements
Source: Dev Growth Differ. 2021 Sep 28;63(7):372–91. doi: 10.1111/dgd.12750 (PMC9293469; doi:10.1111/dgd.12750)
Supplement: Supplementary file 1 — Supplementary Material [file DGD-63-372-s001.pdf]

Supplemental Information

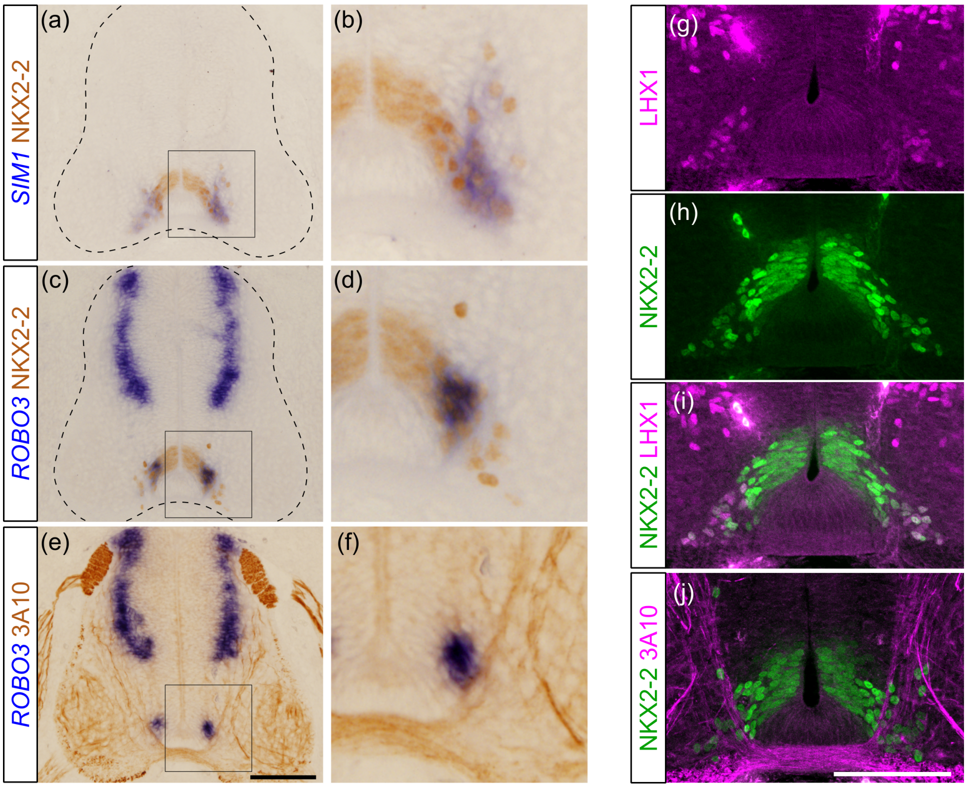

Figure S1 Gene expression profiles in V3 INs of chick embryos

(a–f) Expressions of NKX2-2, *SIM1* and *ROBO3* were examined in the chick spinal cord at the forelimb level at HH25–26. Growing axons were visualized by monoclonal antibody 3A10. Staining of *in situ* hybridization and immunohistochemistry was colored by blue and brown, respectively. b, d and f show the enlarged views of the boxed areas in a, c, and e, respectively. The edges of the neural tube are demarcated by the broken lines. (g–i) Immunohistochemistry using NKX2-2 and LHX1 antibodies. (i) Immunohistochemistry using NKX2-2 and 3A10 antibodies. Ventral-most region of the spinal cord is shown in g–j. Scale bar: 100 µm in e for a, c and e, in j for g–j.

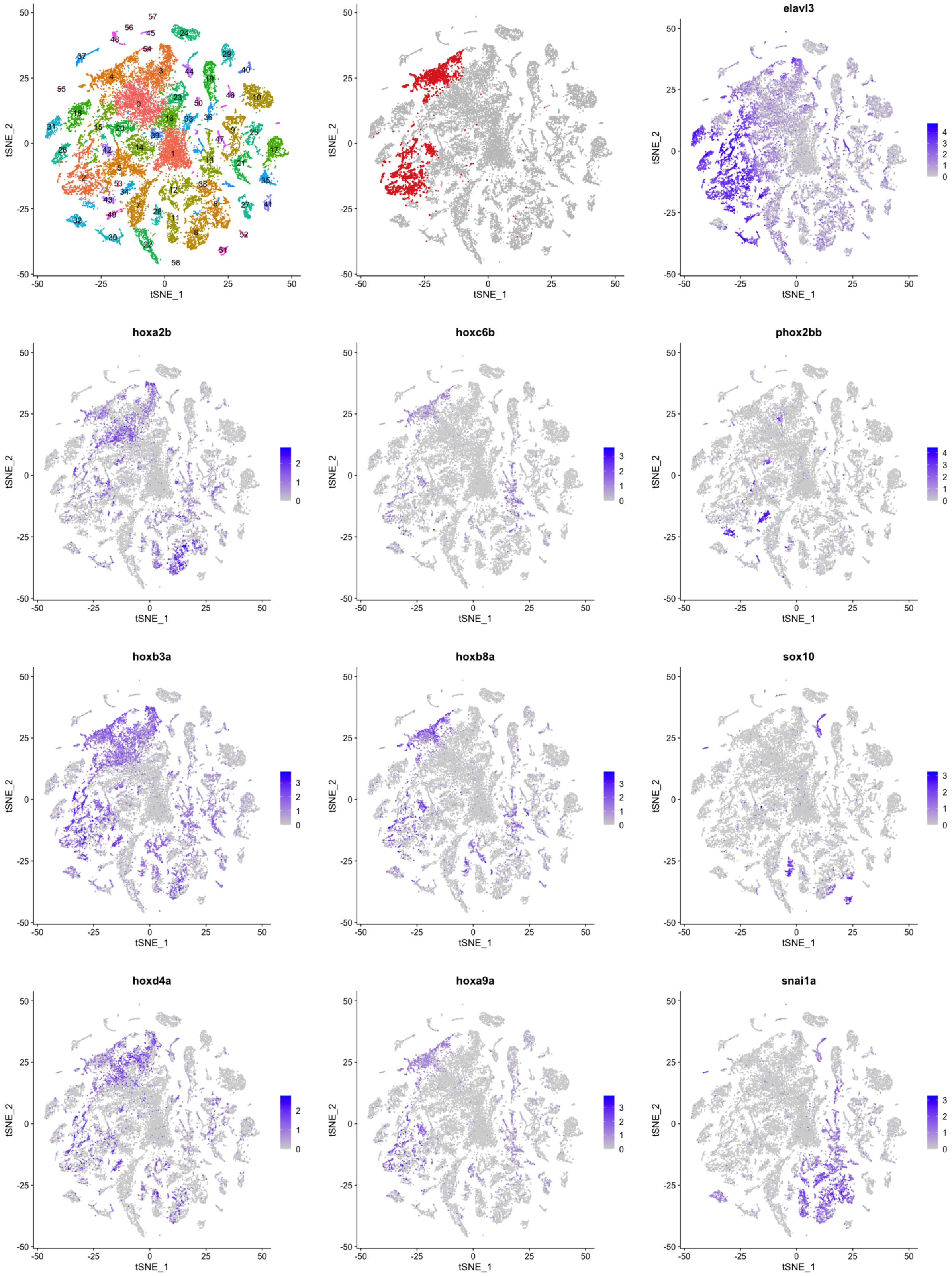

elavl3
hoxa2b
hoxc6b
phox2bb
hoxb3a
hoxb8a
sox10
hoxd4a
hoxa9a
snai1a
tSNE_1
tSNE_2

Figure S2. Subsetting scRNA-seq data derived from zebrafish whole embryo

scRNA-seq data derived from the zebrafish whole embryos at 1 and 2 dpf were analyzed. Clustering results and gene expressions are visualized on the tSNE plot. Top left panel shows distinct clusters labeled with serial numbers 0–58. To define the spinal cord cells (colored red in the top middle panel), several marker genes and *hox* genes are examined. The expression of *elavl3* roughly indicates the neuronal population. Anterior limit of the expression of 3' *hox* genes, such as *hoxa2b*, *hoxb3a*, and *hoxd4a*, are at the hindbrain (Prince et al., 1998a). Whereas *hoxc6b*, *hoxb8a*, and *hoxa9a* is expressed in the spinal cord, but not in the hindbrain (Prince et al., 1998b), indicating that clusters numbered 0, 3, and 15 are likely to be the hindbrain. We also identified the branchiomotor and cranial parasympathetic preganglionic neurons (*phox2bb*), neural crest cells (*sox10*), and mesodermal cells (*snai1a*) to exclude for the downstream analysis. This allow us to define the cells of the spinal cord, which are cluster numbers 2, 4, and 42.

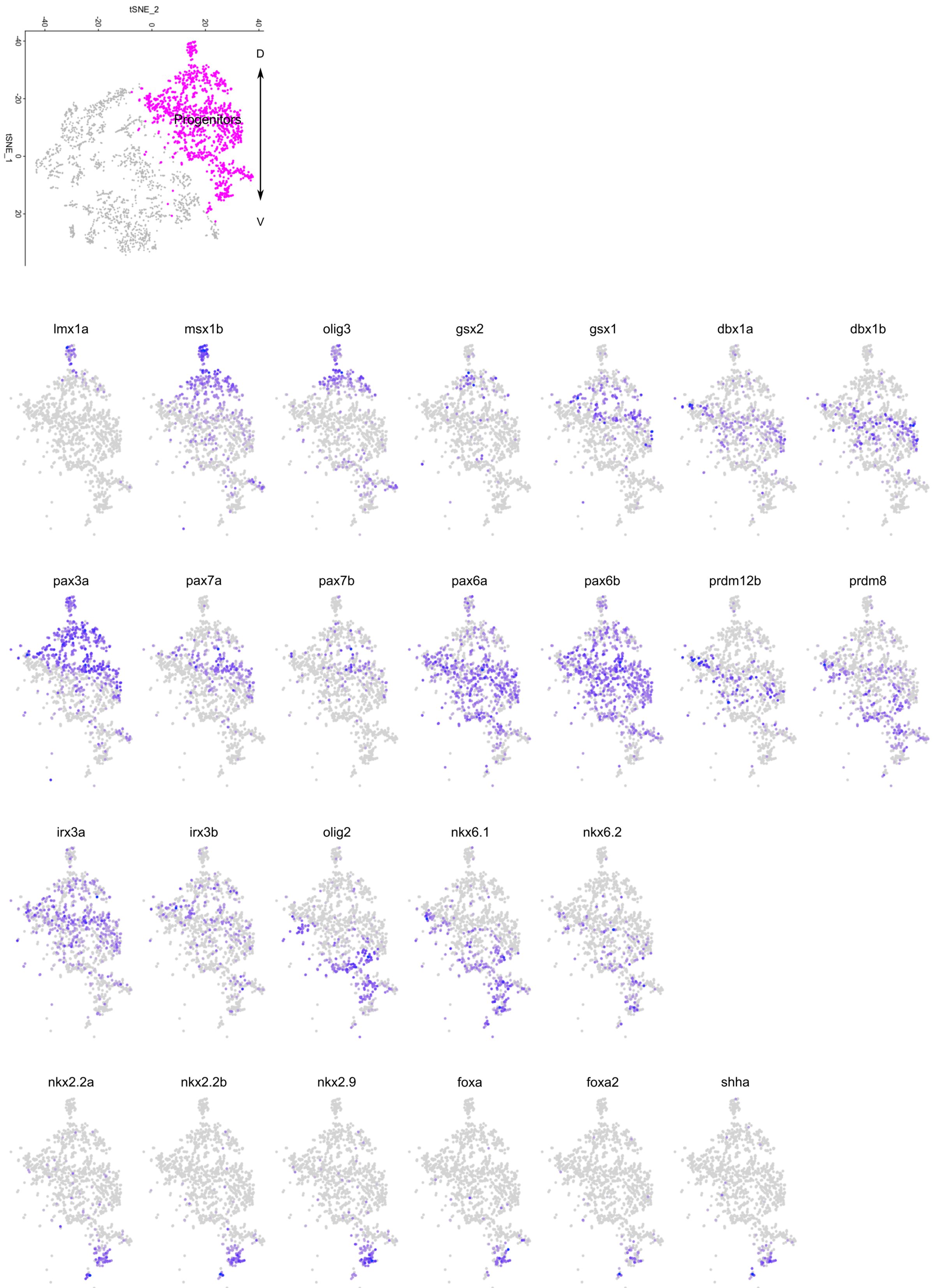
tSNE_2
tSNE_1
Progenitors
D
V
lmx1a
msx1b
olig3
gsx2
gsx1
dbx1a
dbx1b
pax3a
pax7a
pax7b
pax6a
pax6b
prdm12b
prdm8
irx3a
irx3b
olig2
nkx6.1
nkx6.2
nkx2.2a
nkx2.2b
nkx2.9
foxa
foxa2
shha

Figure S3. Gene expression in the progenitor domains of the zebrafish spinal cord

Top panel is identical to the tSNE plot in Figure 2, but is rotated 90 degrees clockwise. All other panels show the expression of domain specific TFs. Only progenitor cells are shown. Cell arrangement in this plot is parallel to that *in vivo* along dorsal-ventral axis. D and V indicate dorsal and ventral, respectively. These data support that progenitor domain organization is highly conserved in vertebrates.

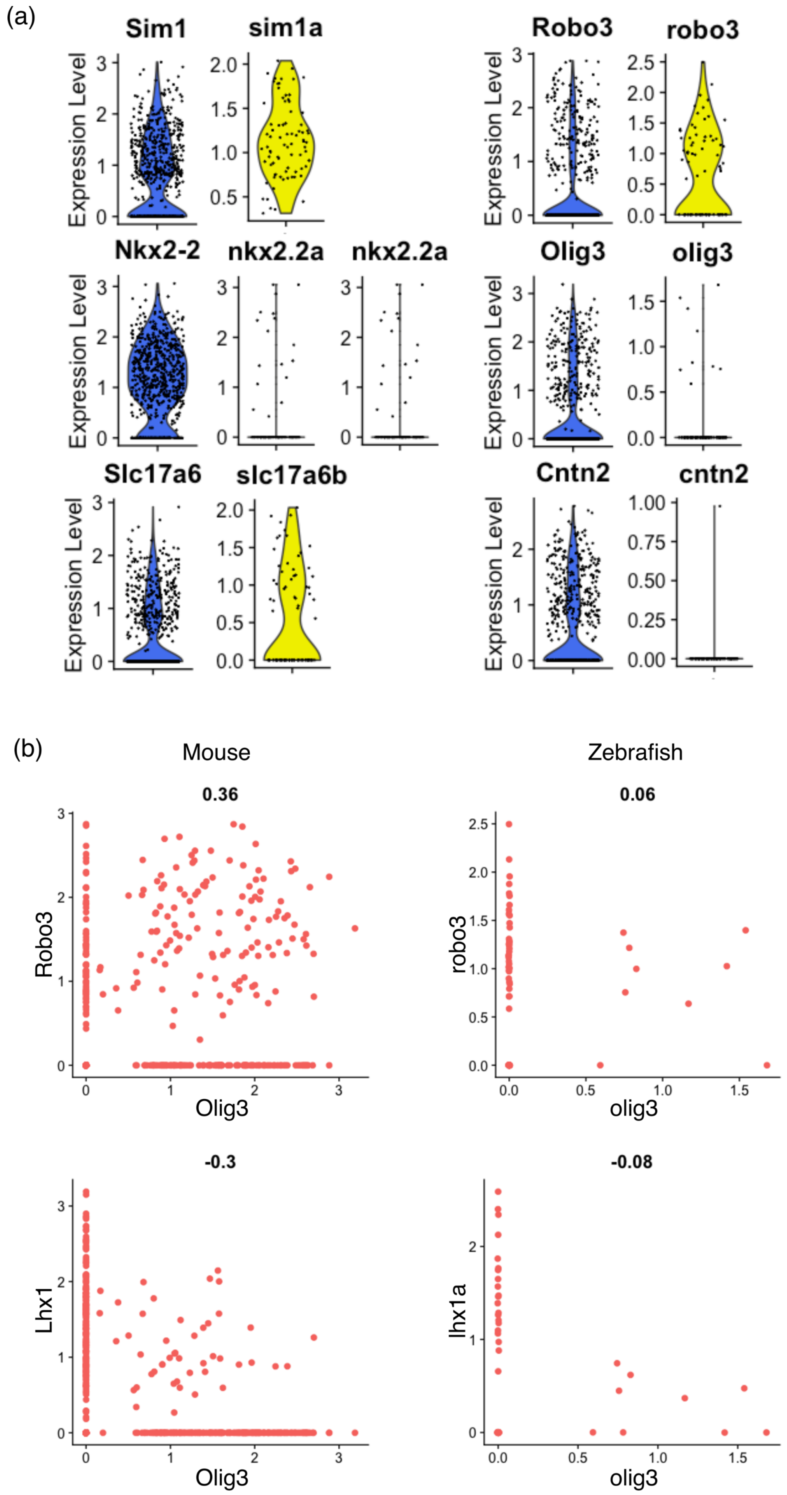

Figure S4. Comparisons of selected marker expression in V3 INs between mice and zebrafish

From the mouse V3 IN dataset, *Sim1*$^{+}$ clusters (1, 2, 3, 4, 5, 7, and 11 in Fig.1) were extracted. From the zebrafish spinal cord dataset, *sim1*$^{+}$ cells were extracted. Then, expression levels of genes indicated are visualized by violin plot (a). Scatter plot of *Olig3-Robo3*, or *Olig3-Lhx1* are shown in (b). Mouse and zebrafish violin plots are colored blue and yellow, respectively. Violin plots indicate differences of gene expression levels in *Nkx2-2*, *Olig3*, and *Cntn2*. In scatter plots, Pearson correlations are provided above the plot. Coexpression of *Olig3* and *Robo3*, as well as mutual exclusive expression of *Olig3* and *Lhx1*, were observed in the mice but not in the zebrafish.

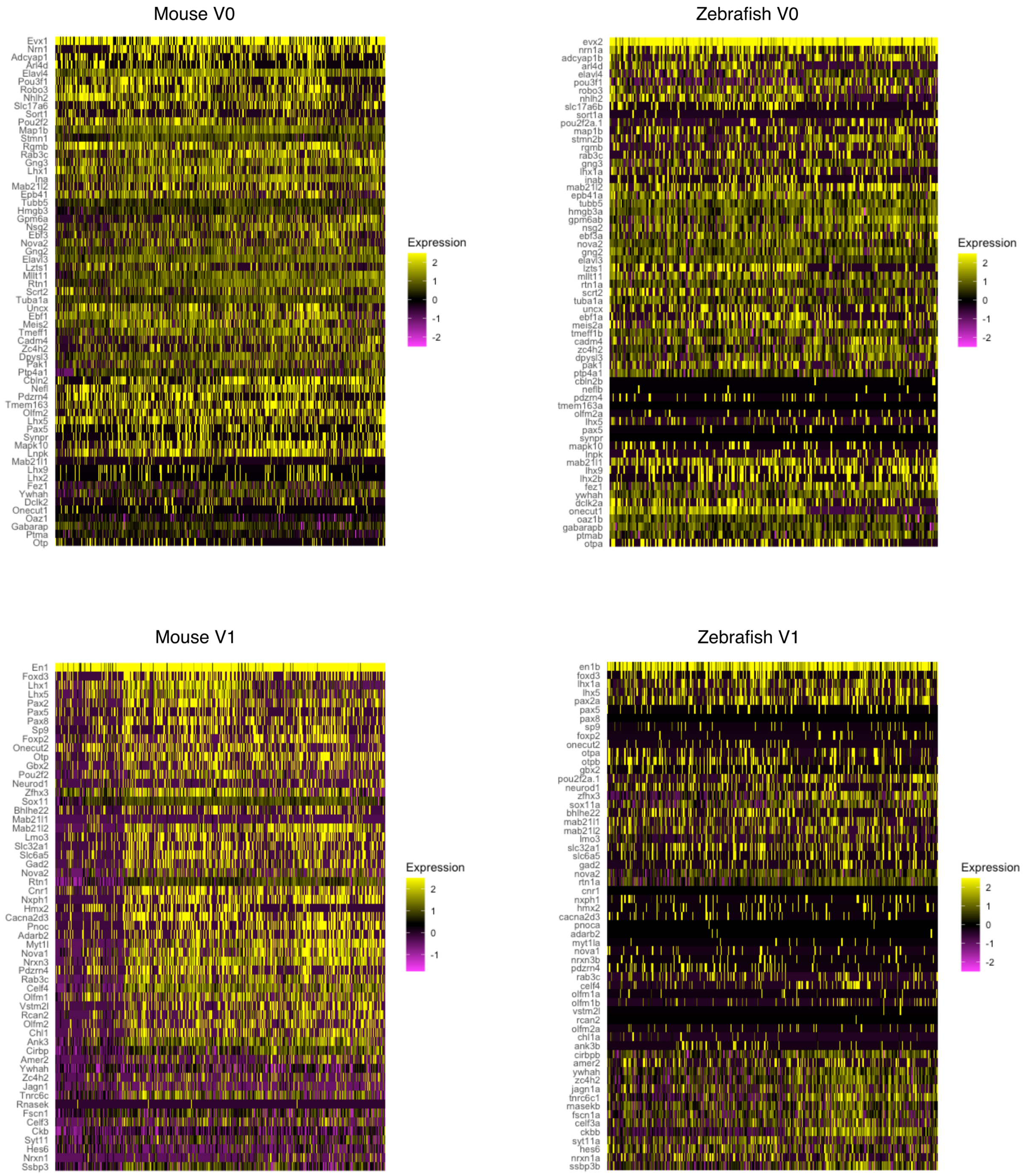

Figure S5. Heatmaps showing differentially expressed genes in V0 and V1 INs

We identified differentially expressed genes (DEGs) between V0 and non-V0 INs, or V1 and non-V1 INs using mouse and zebrafish whole spinal cord datasets independently. The top 50 DEGs from mouse and zebrafish were manually combined, making DEG lists for V0 and V1 INs. Then, expression levels of genes of the DEG list in individual cells were visualized by heatmap. There are many genes showing different expression levels between mice and zebrafish. For example, in V1 INs, *Pax5*, *Pax8*, *Sp9*, *Foxp2*, and *Onecut2* were highly expressed in many cells in the mice, but not in the zebrafish.

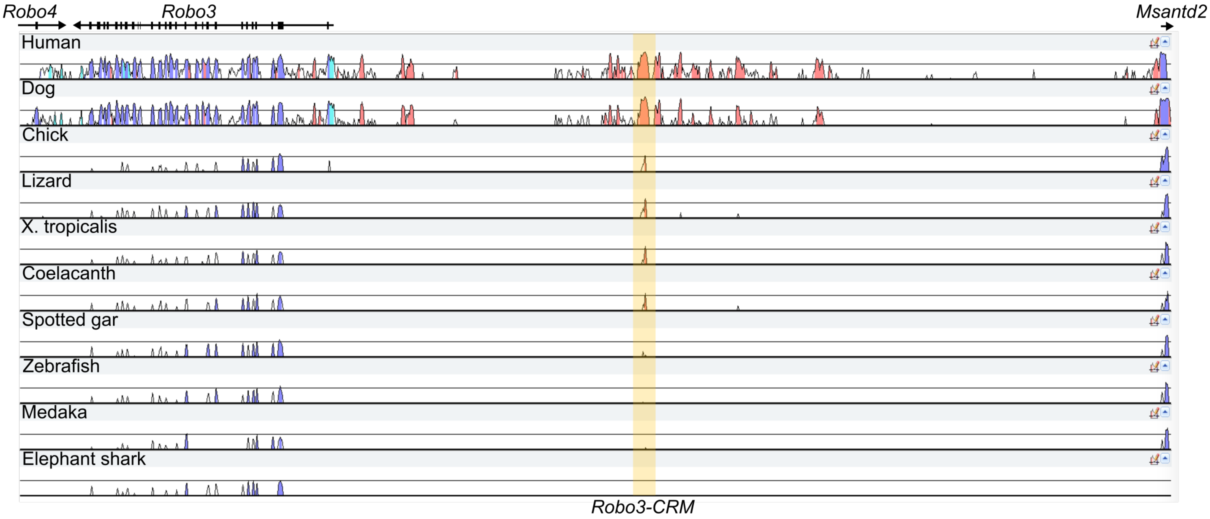

Figure S6. Alignment of *Robo3* locus visualized by VISTA

VISTA plot of *Robo3* locus. Genomic region spanning from *Robo3* to the edge of *Msantd2* were aligned. The base sequence is mouse, and the species compared are indicated on the left side. The peaks of the conserved regions are colored pink (non-coding sequences), dark blue (exons), or light blue (UTRs). *Robo3-CRM* is highlighted in yellow.

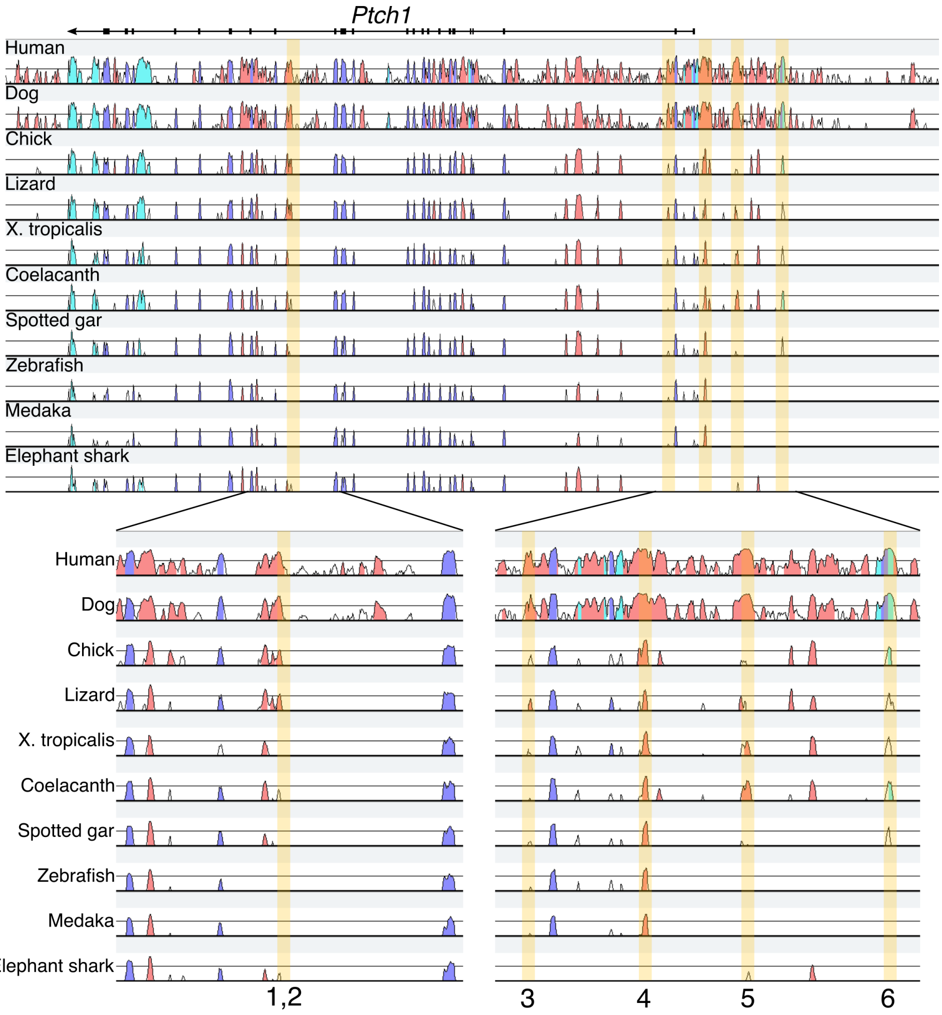

Figure S7. Alignment of *Ptch1* locus visualized by VISTA

VISTA plot of *Ptch1* locus. The aligned genomic region corresponds to Figure 5b. The base sequence is mouse, and the species compared are indicated on the left side. The peaks of conserved regions are colored pink (non-coding sequences), dark blue (exons), or light blue (UTRs). The highlighted regions (GBSs) and labeled numbers correspond to Figure 5b. Enlarged views of the highlighted regions are displayed in the bottom panels. We checked the GBS sequences in these regions and confirmed the extent of conservation. GBS 1 and 2 are partially overlapping sequences, and are not conserved in any of the species examined. GBS 3 and 4 are conserved in the bony vertebrates, but not in the elephant shark. GBS 5 is conserved in the tetrapods, spotted gar and elephant shark, but not in the teleosts. GBS 6 is conserved in the tetrapods and spotted gar.

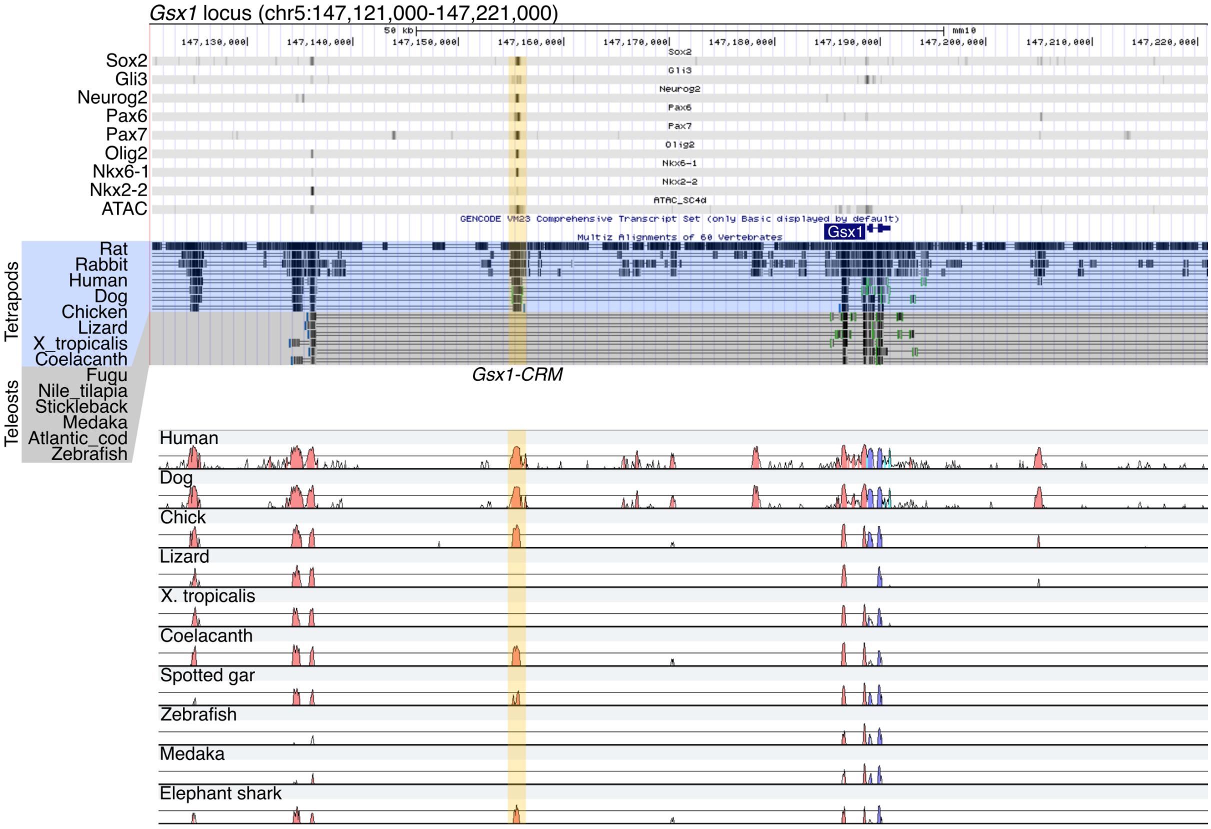

Figure S8. Identification of *Gsx1-CRM* and its diversification in vertebrates

(Top) ChIP-seq and ATAC-seq peak call results are displayed in UCSC genome browser with Multiz Alignments track (display mode is dense). The mouse *Gsx1* locus is displayed, and the region harboring multiple TF binding sites (*Gsx1-CRM*) is highlighted in yellow. (Bottom) The same genomic regions from several species are aligned, and sequence conservation is visualized by VISTA. The base sequence is mouse, and the species compared are indicated on the left side. The peaks of the conserved regions are colored pink (non-coding sequences), dark blue (exons), or light blue (UTRs). *Gsx1-CRM* are not conserved in the teleosts. Conservation of *Gsx1-CRM* in the lizard and *X. tropicalis* was detected by Multiz alignment, but not by VISTA. The reason for this discrepancy is unclear, but possibly due to the different algorithms.

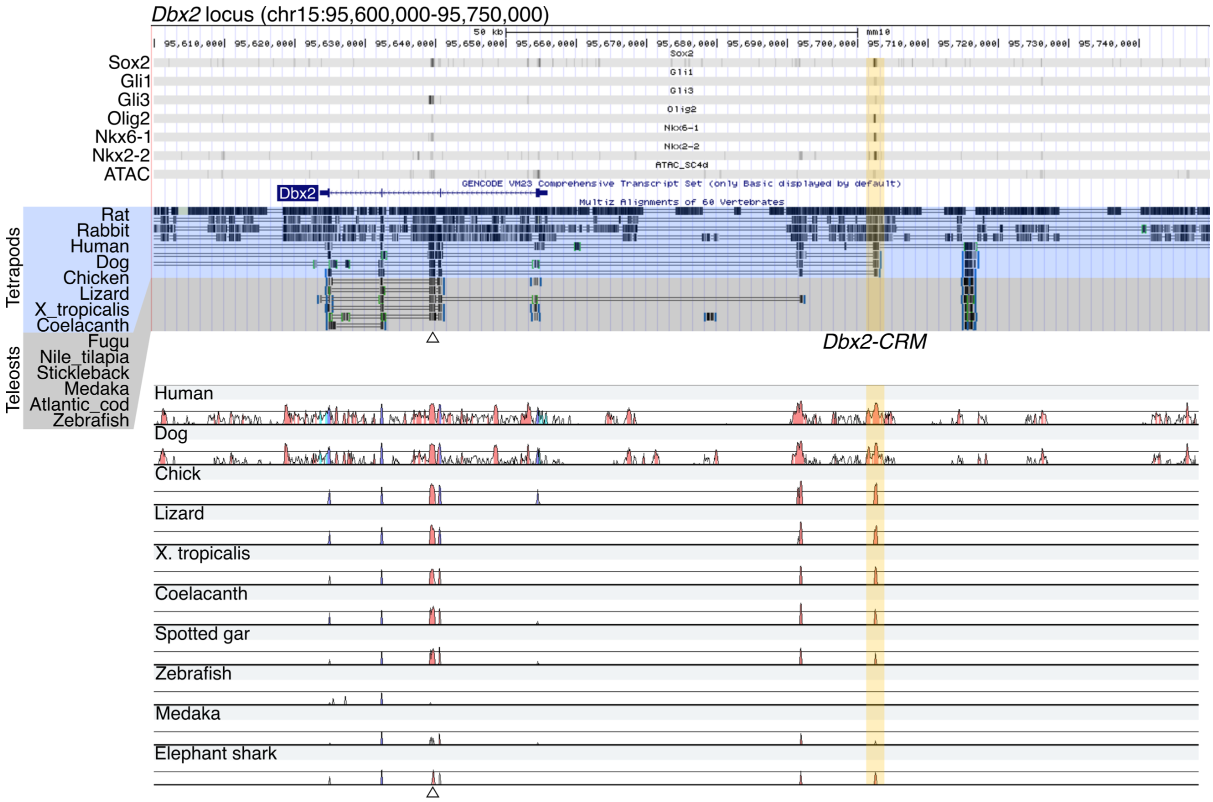

Figure S9. Identification of *Dbx2-CRM* and its diversification in vertebrates

(Top) ChIP-seq and ATAC-seq peak call results are displayed in UCSC genome browser with Multiz Alignments track (display mode is dense). The mouse *Dbx2* locus is displayed, the region harboring multiple TF binding sites (*Dbx2-CRM*) is highlighted in yellow, and previously validated CRM is indicated by an open triangle under the track (Oosterveen et al., 2012). (Bottom) The same genomic regions from several species are aligned and sequence conservation is visualized by VISTA. The base sequence is mouse, and the species compared are indicated on the left side. The peaks of the conserved regions are colored pink (non-coding sequences), dark blue (exons), or light blue (UTRs).

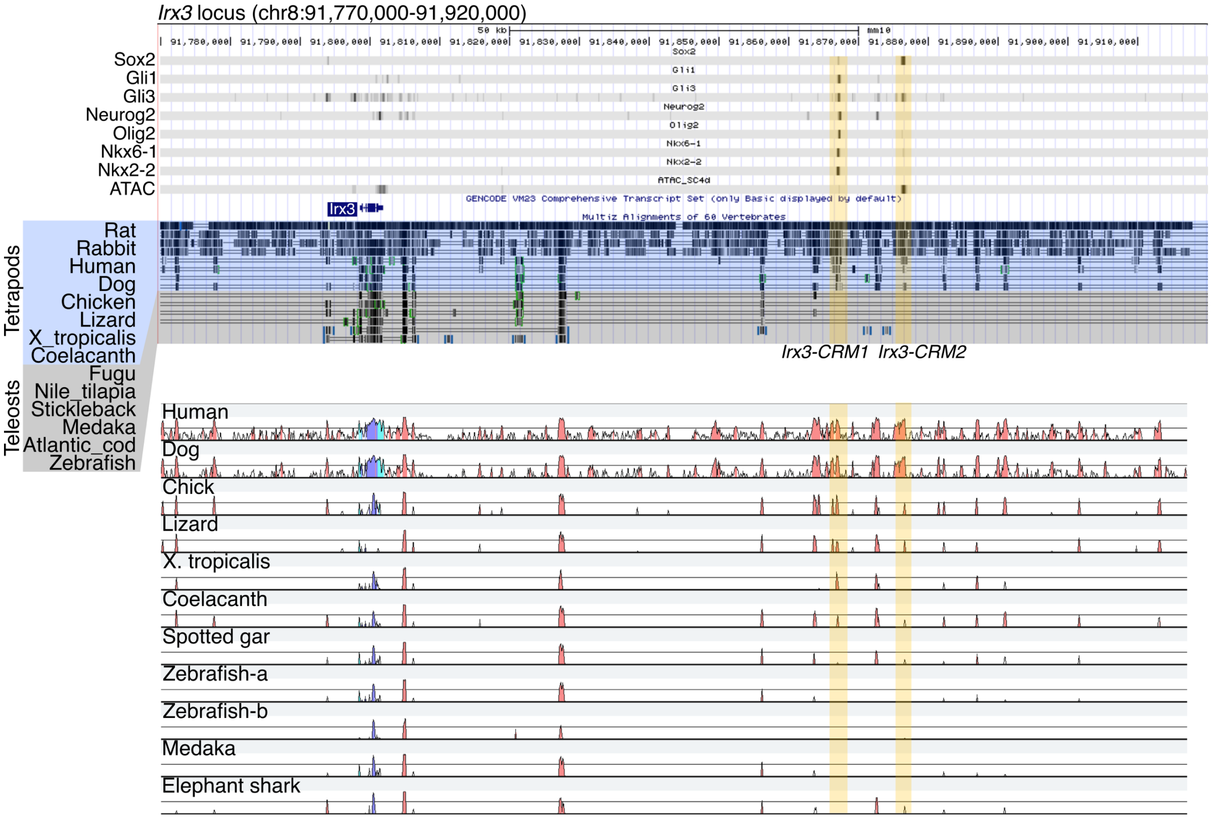

Figure S10. Identification of *Irx3-CRM* and its diversification in vertebrates

(Top) ChIP-seq and ATAC-seq peak call results are displayed in UCSC genome browser with Multiz Alignments track (display mode is dense). The mouse *Irx3* locus is displayed, and the regions harboring multiple TF binding sites (*Irx3-CRM1* and *Irx3-CRM2*) are highlighted in yellow. (Bottom) The same genomic regions from several species are aligned and sequence conservation is visualized by VISTA. The base sequence is mouse, and the species compared are indicated on the left side. The peaks of the conserved regions are colored pink (non-coding sequences), dark blue (exons), or light blue (UTRs). Zebrafish possess two *irx3* genes (*irx3a* and *irx3b*), thus both loci are included in the alignments (Zebrafish-a and Zebrafish-b correspond to *irx3a* and *irx3b* locus, respectively).

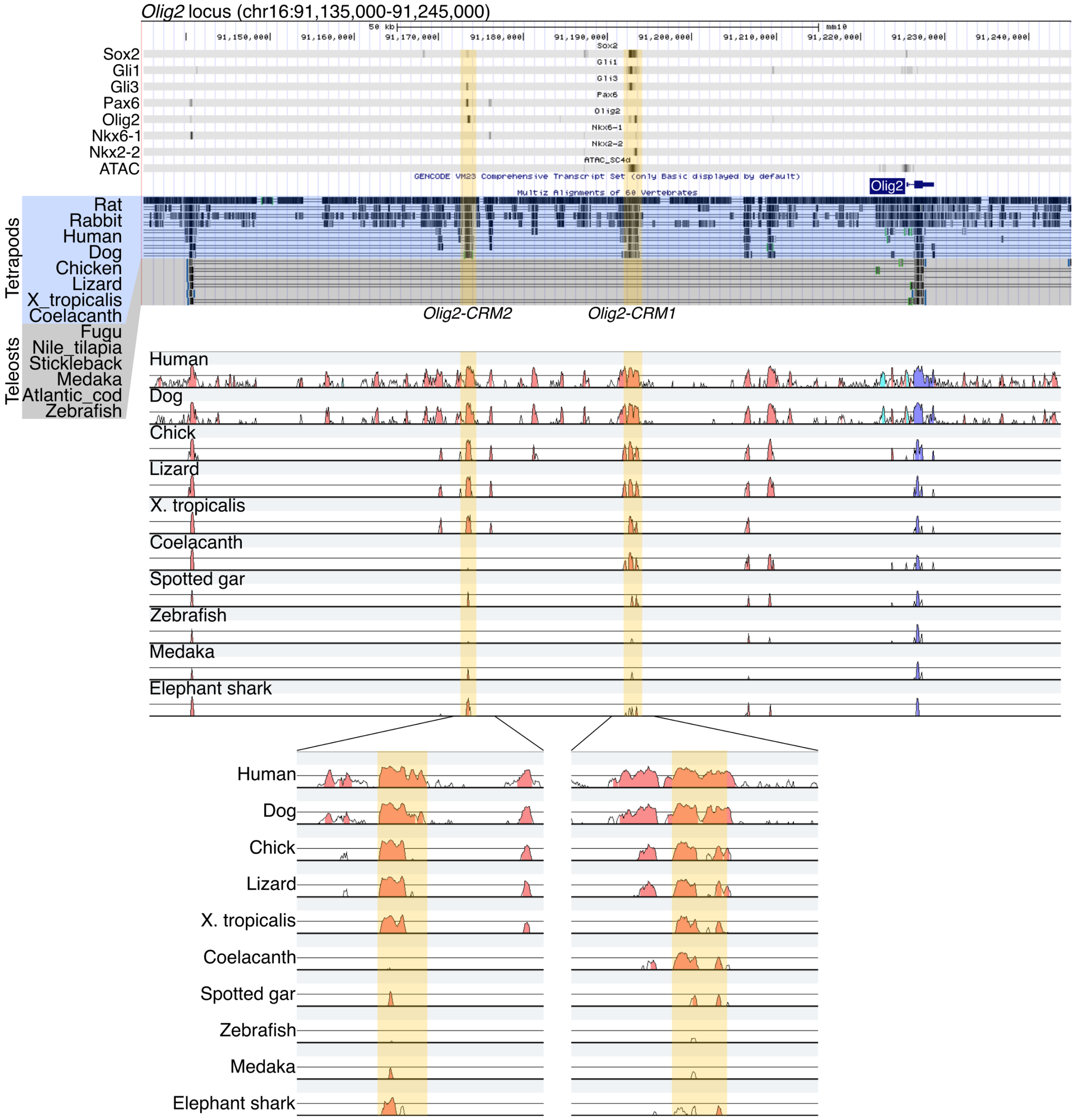

Figure S11. Identification of *Olig2-CRM* and its diversification in vertebrates

(Top) ChIP-seq and ATAC-seq peak call results are displayed in UCSC genome browser with Multiz Alignments track (display mode is dense). The mouse *Olig2* locus is displayed, and the regions harboring multiple TF binding sites (*Olig2-CRM1* and *Olig2-CRM2*) are highlighted in yellow. *Olig2-CRM1* was previously validated (Exelby et al., 2021; Oosterveen et al., 2012; Peterson et al., 2012; Wang et al., 2011). (Bottom) The same genomic regions from several species are aligned and sequence conservation is visualized by VISTA. The base sequence is mouse, and the species compared are indicated on the left side. The peaks of the conserved regions are colored pink (non-coding sequences), dark blue (exons), or light blue (UTRs). Enlarged views of the plots focusing on CRMs are displayed below.

Table S2
Sequences of primers used for cDNA amplification for RNA probe preparation

chick ROBO3 Forward	TCCAACTCCTCCGAGCTGCTGCTCGGC
chick ROBO3 Reverse	ATGGAGATGGGCGCACTGCGAGCACCC
chick SIM1 Forward	TTAAGAATTCGCGGGGGCTACAAGGTCATCC
chick SIM1 Reverse	ATATAAGCTTGGGGAGAGGCTGTGTCGGTGAGG
chick GSX1 Forward	AGCTGAATTCCTTCCTGGTGGACTCGCTG
chick GSX1 Reverse	GTACAAGCTTCAAAATCATCCGGCTGCACG
chick DBX2 Forward	AGCTGAATTCCAGCCTGGGCAAAAGTTTCC
chick DBX2 Reverse	GTACAAGCTTGCTCTTGGAGGTGGTTGTGT
chick IRX3 Forward	AGCTGAATTCAGTACATCAGGCCGCTGTACCC
chick IRX3 Reverse	GTACAAGCTTTCCCTCTTGTCCTCTTCCCCCT
mouse Robo3 Forward	AATTGAATTCCTACCAGGCCAGATCTATCGAGCC
mouse Robo3 Reverse	AATTGTCGACGGGGGTAAGAGTAGCTGTGGACTG
mouse Sim1 Forward	AATTGAATTCATAGGCAGTTCCCAGATCGCAGTT
mouse Sim1 Reverse	AATTGGATCCGCCATTGCAGCCCAAGGAATAGTT

Table S3
CRM positions examined in this study

CRM	Position in mouse reference genome (mm10)
Robo3-CRM	chr9:37,453,722-37,454,773
Pax6-CRM	chr2:105,611,719-105,612,835
Gsx1-CRM	chr5:147,155,012-147,156,180
Dbx2-CRM	chr15:95,701,894-95,703,059
Irx3-CRM1	chr8:91,866,617-91,867,812
Irx3-CRM2	chr8:91,875,790-91,876,952
Olig2-CRM2	chr16:91,172,997-91,174,126

Table S4
Antibodies used in this study

Antibody	Source	ID
Mouse anti-Neurofilament-associated antigen	Developmental studies hybridoma bank (DSHB)	3A10
Mouse anti-Nkx2-2	DSHB	74.5A5
Mouse anti-Lhx1	DSHB	4F2
Mouse anti-Isl1/2	DSHB	39.4D5
Mouse anti-Evx1/2	DSHB	99.1-3A2
Mouse anti-Pax6	DSHB	PAX6
Rabbit anti-Olig2	Millipore	AB9610
Mouse anti-GFP	DSHB	DSHB-GFP-4C9
Rabbit anti-GFP	MBL	598
Rabbit anti-GFP, Alexa Fluor 488 conjugated	Thermo Fisher	A21311
Alexa Fluor 488 anti-mouse IgG1	Thermo Fisher	A21121
Alexa Fluor 488 anti-mouse IgG2b	Thermo Fisher	A21141
Alexa Fluor 488 anti-rabbit IgG	Thermo Fisher	A11008
Alexa Fluor 594 anti-mouse IgG1	Thermo Fisher	A21125
Alexa Fluor 647 anti-mouse IgG1	Thermo Fisher	A21240
Alexa Fluor 647 anti-mouse IgG2b	Thermo Fisher	A21242
Alexa Fluor Plus 647 anti-rabbit IgG	Thermo Fisher	A32733
Goat anti-rabbit IgG, biotinylated	Vector laboratories	BA-1000
Horse anti-mouse IgG, biotinylated	Vector laboratories	BA-2000
Anti-DIG-AP	Sigma-Aldrich	11093274910

Table S5
Summary of the results of *in ovo* electroporation

Names of plasmid electroporated	Plasmid conc.	Fixed stage	GFP expression* (number of embryos observed)	Displayed in Fig.
Robo3-CRM::GFP	2μg/μL	HH20	+ (3)	Displayed in Fig.4
	2μg/μL	HH25	+ (4)	Displayed in Fig.4
Pax6-CRM::GFP	2μg/μL	HH19–20	++ (6)	
	0.5μg/μL	HH19–20	++ (7)	
	0.1μg/μL	HH19–20	++ (3)	
	50ng/μL	HH19–20	++ (5)	
	30–40ng/μL	HH19–20	+ (6)	Displayed in Fig.7
	20ng/μL	HH19–20	+ (3)	
	15ng/μL	HH19–20	+ (11)	
	5ng/μL	HH19–20	– (7)	
Gsx1-CRM::GFP	0.5μg/μL	HH19–20	+ (5)	
	0.1μg/μL	HH19–20	++ (6), + (4)	
	40ng/μL	HH19–20	+ (6)	Displayed in Fig.7
	30ng/μL	HH19–20	+ (9)	
	20ng/μL	HH19–20	+ (6)	
Dbx2-CRM::GFP	2μg/μL	HH19–20	+ (8)	
	0.1μg/μL	HH19–20	+ (4)	
	50ng/μL	HH19–20	+ (5)	Displayed in Fig.7
Irx3-CRM1::GFP	4μg/μL	HH19–20	– (3)	
	2μg/μL	HH19–20	– (5)	
Irx3-CRM2::GFP	0.5μg/μL	HH19–20	++ (7)	
	0.1μg/μL	HH19–20	++ (4)	
	50ng/μL	HH19–20	+ (4)	
	40ng/μL	HH19–20	++ (4), + (7)	Displayed in Fig.7
	30ng/μL	HH19–20	+ (4)	
	20ng/μL	HH19–20	– (5)	
Olig2-CRM2::GFP	2μg/μL	HH19–20	++ (3)	
	0.1μg/μL	HH19–20	+ (12)	
	50ng/μL	HH19–20	+ (8), – (2)	
	40ng/μL	HH19–20	+ (5)	Displayed in Fig.7
	20ng/μL	HH19–20	– (5)	

* GFP expressions are represented as follows; ++: non-specific (ubiquitous) expression (GFP expression completely overlap with mCherry expression), +: regionally restricted expression, –: weak or no expression.

References

Prince, V. E., Moens, C. B., Kimmel, C. B., & Ho, R. K. (1998a). Zebrafish hox genes: expression in the hindbrain region of wild-type and mutants of the segmentation gene, valentino. *Development*, *125*, 393–406.

Prince, V. E., Joly, L., Ekker, M., & Ho, R. K. (1998b). Zebrafish hox genes: genomic organization and modified colinear expression patterns in the trunk. *Development*, *125*, 407–420.
